# Hippo pathway affects survival of cancer patients: extensive analysis of TCGA data and review of literature

**DOI:** 10.1038/s41598-018-28928-3

**Published:** 2018-07-13

**Authors:** Anello Marcello Poma, Liborio Torregrossa, Rossella Bruno, Fulvio Basolo, Gabriella Fontanini

**Affiliations:** 1 Department of Surgical, Medical, Molecular Pathology and Critical Area, University of Pisa, Pisa, Italy; 2 Department of Laboratory Medicine, Section of Pathology, University Hospital of Pisa, Pisa, Italy

## Abstract

The disruption of the Hippo pathway occurs in many cancer types and is associated with cancer progression. Herein, we investigated the impact of 32 Hippo genes on overall survival (OS) of cancer patients, by both analysing data from The Cancer Genome Atlas (TCGA) and reviewing the related literature. mRNA and protein expression data of all solid tumors except pure sarcomas were downloaded from TCGA database. Thirty-two Hippo genes were considered; for each gene, patients were dichotomized based on median expression value. Survival analyses were performed to identify independent predictors, taking into account the main clinical-pathological features affecting OS. Finally, independent predictors were correlated with YAP1 oncoprotein expression. At least one of the Hippo genes is an independent prognostic factor in 12 out of 13 considered tumor datasets. mRNA levels of the independent predictors coherently correlate with YAP1 in glioma, kidney renal clear cell, head and neck, and bladder cancer. Moreover, literature data revealed the association between YAP1 levels and OS in gastric, colorectal, hepatocellular, pancreatic, and lung cancer. Herein, we identified cancers in which Hippo pathway affects OS; these cancers should be candidates for YAP1 inhibitors development and testing.

## Introduction

Since its discovery in *Drosophila Melanogaster*^1^, Hippo pathway has gained ever-increasing attention. Nowadays, the involvement of Hippo pathway in cancer development and progression is well recognised. However, the different and sometimes controversial roles that it may play rise the scientific interest about this pathway. The main example is the enhanced immune response against the tumor after depletion of the LATS1-2 oncosuppressors observed in immune-competent mice^2^. Nevertheless, the canonical oncosuppressor role is the widely accepted one^3,4^. In this view, the kinases axis, represented by STK3-4/LATS1-2, works as a brake, controlling cell cycle, apoptosis and cell patterning, thus avoiding uncontrolled proliferation and loss of epithelial-like features. LATS kinases can be activated by a great variety of stimuli through different groups of kinases, such as MAP4Ks and TAOKs^3^. The activity of these kinases depends on the presence of co-activators, among which SAV1, NF2 and FRMD6 represents the first to be discovered^1,5^.

**Figure 1:**
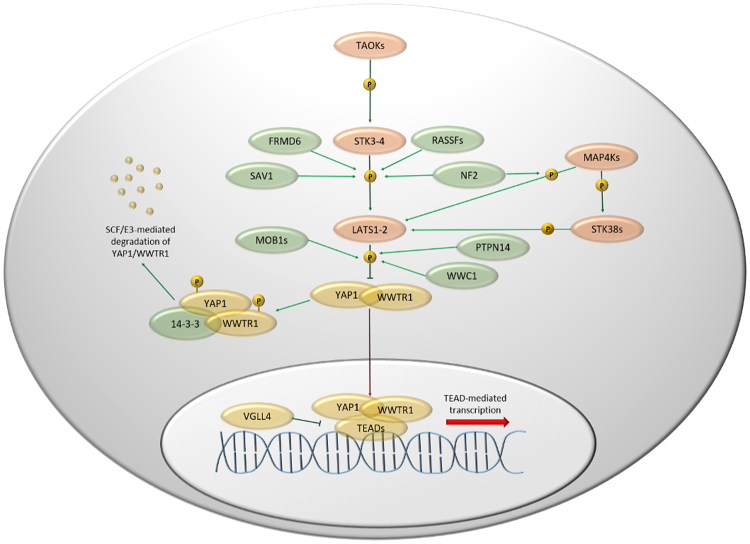
Hippo pathway. In orange are kinases, in green coactivators or scaffold proteins and in yellow transcription factors or proteins interacting with transcription factors. Green lines refer to active Hippo pathway, which leads to YAP1-WWTR1 inactivation; red lines relate the TEAD-mediated transcription, when the pathway is inactive.

The final outcome of Hippo pathway is the LATS-mediated phosphorylation of YAP1, mainly at the residue S127, leading to its cytoplasmic retention and eventually degradation^6^. Unphosphorylated YAP1, together with WWTR1, activates the TEAD1-4-mediated transcription in the nucleus, representing the cancer progression accelerator. Finally, VGLL4 is a peptide acting as an oncosuppressor by competing with YAP1-WWTR1 complex to TEADs binding^3^ (Fig. 1). The presence of natural YAP1 competitor uncovered a new scenario to counterbalance the insufficient Hippo pathway oncosuppressor activity. Several molecules are capable to interfere with YAP1 activity by both mimicking VGLL4 function and preventing YAP1-WWTR1 interaction^7^. Among YAP1 inhibitors, the photosensitizer verteporfin, already approved by the Food and Drug Administration for the macular degeneration treatment, showed excellent results both *in vitro* and in mice, with no or limited side effects^8,9^. Verteporfin is then one of the main candidate to move a step forward as a therapeutic agent for YAP1 inhibition. In the present study, we conducted a data analysis of all solid tumor datasets of The Cancer Genome Atlas (TCGA) except pure sarcomas, and a review of literature to investigate the impact of the Hippo pathway dysregulation on survival of cancer patients, providing food for thought and data-driven proposals for approaching future Hippo-directed therapies.

## Results

### Power analysis and definitive datasets

Thirteen of the twenty-nine downloaded TCGA datasets had β above 0.8 with the set parameters and were selected for further analyses. Details and covariates for each dataset were reported in Table 1.

**Table 1:** Results of power analysis.

Dataset	TCGA id	Sample size	Probability of the event	β (RR = 2.3)	Covariates
**Ovarian Serous Cystadenocarcinoma**	OV	290	0.5655	0.9990	grade, age, clinical stage
**Kidney Renal Clear Cell Carcinoma**	KIRC	520	0.3058	0.9987	pathologic tumor stage
**Head and Neck Squamous Cell Carcinoma**	HNSC	477	0.3333	0.9987	tobacco smoking indicator, age, clinical stage
**Lung Squamous Cell Carcinoma**	LUSC	469	0.3220	0.9980	pathologic stage, age
**Skin Cutaneous Melanoma**	SKCM	393	0.3359	0.9948	pathologic tumor stage
**Lung Adenocarcinoma**	LUAD	468	0.2543	0.9903	pathologic tumor stage, age
**Bladder Urothelial Carcinoma**	BLCA	389	0.2751	0.9826	pathologic tumor stage, age, grade
**Glioblastoma**	GBM	156	0.6795	0.9820	age
**Brain Lower Grade Glioma**	LGG	505	0.1822	0.9653	age, grade
**Liver Hepatocellular Carcinoma**	LIHC	285	0.2351	0.8962	pathologic tumor stage, grade, vascular invasion
**Cervical Squamous Cell Carcinoma and Endocervical Adenocarcinoma**	CESC	279	0.2151	0.8616	clinical stage
**Mesothelioma**	MESO	84	0.6667	0.8384	pathologic stage
**Pancreatic Adenocarcinoma**	PAAD	157	0.3503	0.8300	pathologic tumor stage, residual tumor
Esophageal Carcinoma	ESCA	137	0.3285	0.7502	
Colorectal Adenocarcinoma	COADRED	323	0.1331	0.7323	
Uterine Carcinosarcoma	UCS	55	0.5636	0.5860	
Breast Invasive Carcinoma	BRCA	759	0.0395	0.5777	
Kidney Renal Papilllary Cell Carcinoma	KIRP	239	0.1130	0.5334	
Adrenocortical Carcinoma	ACC	72	0.3056	0.4554	
Cholangiocarcinoma	CHOL	34	0.4412	0.3321	
Uterine Corpus Endometrial Carcinoma	UCEC	172	0.0756	0.2948	
Uveal Melanoma	UVM	79	0.1646	0.2923	
Thyroid Carcinoma	THCA	435	0.0253	0.2565	
Prostate Adenocarcinoma	PRAD	496	0.0161	0.1986	
Kidney Chromophobe	KICH	63	0.1111	0.1777	
Pheochromocytoma and Paraganglioma	PCPG	178	0.0337	0.1598	
Thymoma	THYM	117	0.0513	0.1591	
Stomach Adenocarcinoma	STAD	15	0.3333	0.1349	
Testicular Germ Cell Cancer	TGCT	131	0.0153	0.0802	

In bold are datasets with β above 0.8 that were selected for further analyses. For these datasets, clinical-pathological covariates affecting patients’ survival according to the eighth edition of the American Joint Committee on Cancer are listed. RR, postulated risk ratio.

### Survival analyses

**Figure 2:**
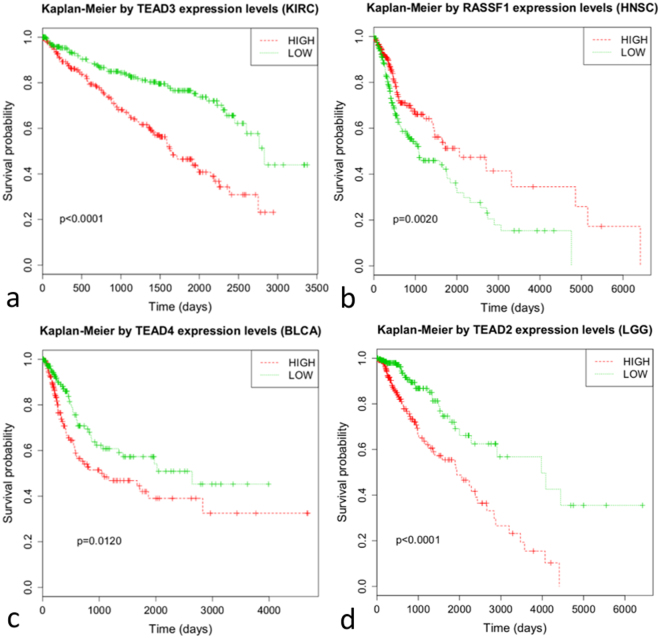
Kaplan-Meier curves. In the panel are Kaplan-Meier curves of the four independent predictors that correlated with YAP1 protein, coherently with the canonical role of the Hippo pathway. In detail: (**a**) *TEAD3* in Kidney Renal Clear Cell Carcinoma; (**b**) *RASSF1* in Head and Neck Squamous Cell Carcinoma; (**c**) *TEAD4* in Bladder Urothelial Carcinoma; (**d**) *TEAD2* in Brain Lower Grade Glioma. The log-rank p values are also reported.

Univariate and multivariate results were summarized in Table 2, p values of univariate and multivariate analyses were reported in Supplementary Tables S1 and S2 respectively. Briefly, univariate analyses showed that 12 out of 13 cancer models had at least one Hippo gene associated with patients prognosis and ten datasets had 3 or more significant genes. Brain lower grade glioma and kidney renal clear cell carcinoma had the higher number of Hippo genes associated with patients’ survival, 16 and 15 respectively, whereas liver hepatocellular carcinoma was the only dataset with no significant genes. With regard to genes, *TEAD4* and *LATS2* were the most frequently associated with patients’ survival, in 6 and 5 out of 13 datasets respectively. Genes and clinical-pathological parameters resulting associated with prognosis after univariate analyses were then used in the multivariate cox regression. Again, 12 out of 13 datasets had at least one Hippo gene as independent survival predictor, and *TEAD4* resulted an independent prognostic factor in 3 different datasets. Survival curves of the independent predictors are reported in Fig. 2 and in Supplementary Figure S1.

**Table 2:** Results of univariate and multivariate analyses.

Dataset	Prognostic factor	Independent prognostic factor	Hazard ratio (95% CI)	Dataset	Prognostic factor	Independent prognostic factor	Hazard ratio (95% CI)
OV	*MAP4K2*	yes	0.71 (0.52–0.97)	LGG	*LATS2*	no	
	age (58 years)	no			*MAP4K1*	no	
KIRC	*FRMD6*	no			*MOB1A*	no	
	*LATS1*	no			*MOB1B*	no	
	*LATS2*	no			*NF2*	no	
	*MAP4K1*	no			*RASSF1*	no	
	*PTPN14*	no			*STK3*	no	
	*RASSF1*	no			*STK38*	no	
	*RASSF6*	no			*STK4*	no	
	*SAV1*	no			*TAOK2*	no	
	*TAOK1*	no			*TEAD2*	yes	0.55 (0.31–0.98)
	*TAOK3*	yes	1.66 (1.13–2.45)		*TEAD3*	no	
	*TEAD1*	no			*TEAD4*	no	
	*TEAD3*	yes	0.69 (0.47–0.99)		*VGLL4*	no	
	*TEAD4*	no			*WWTR1*	no	
	*TNIK*	no			*YAP1*	no	
	*WWTR1*	yes	1.78 (1.09–2.89)		age (41 years)	yes	5.16 (3.00–8.90)
	pathologic tumor stage	yes	stage III: 2.40 (1.52–3.78); stage IV: 6.81 (4.41–10.51)		grade	yes	G3: 2.54 (1.47–4.41)
HNSC	*MAP4K1*	no		CESC	*LATS1*	no	
	*RASSF1*	yes	1.61 (1.13–2.31)		*LATS2*	yes	0.40 (0.23–0.72)
	*TAOK2*	no			*MAP4K1*	yes	1.80 (1.05–3.08)
	*WWTR1*	no			*TNIK*	no	
LUSC	*LATS2*	no			clinical stage	yes	stage IV: 2.43 (1.18–5.02)
	*MAP4K2*	yes	0.63 (0.45–0.88)				
	*MAP4K5*	no		MESO	*FRMD6*	no	
	*MINK1*	yes	0.70 (0.50–0.97)		*MAP4K4*	yes	0.45 (0.23–0.88)
	*WWC1*	no			*RASSF6*	no	
SKCM	*PTPN14*	yes	0.66 (0.46–0.95)		*SAV1*	yes	2.42 (1.28–4.58)
	*TAOK3*	no			*STK38L*	no	
	*TEAD4*	yes	0.69 (0.48–0.97)		*TAOK3*	no	
	pathologic tumor stage	no			*TNIK*	no	
LUAD	*FRMD6*	yes	0.66 (0.45–0.96)	PAAD	*VGLL4*	no	
	*LATS2*	no			*FRMD6*	no	
	*TEAD4*	no			*MAP4K4*	no	
	pathologic tumor stage	yes	stage II: 2.40 (1.50–3.83); stage III: 3.83 (2.39–6.14); stage IV: 3.82 (1.93–7.56)		*MOB1A*	no	
BLCA	*TEAD4*	yes	0.66 (0.44–0.97)		*NF2*	no	
	age (69 years)	yes	1.61 (1.09–2.37)		*PTPN14*	no	
	pathologic tumor stage	yes	stage III: 2.10 (1.13–3.92); stage IV: 3.80 (2.11–6.86)		*SAV1*	no	
GBM	*MAP4K2*	no			*STK3*	no	
	*RASSF1*	no			*TAOK2*	no	
	*TEAD2*	yes	1.73 (1.16–2.58)		*TEAD4*	yes	0.40 (0.22–0.75)
	*TNIK*	yes	1.52 (1.01–2.29)		*YAP1*	no	
LIHC	pathologic tumor stage	yes	stage IV: 5.21 (1.58–17.19)		pathologic tumor stage	no	
	vascular invasion	yes	micro: 0.36 (0.14–0.92); none: 0.36 (0.16–0.81)		residual tumor	yes	R1: 3.03 (1.57–5.85)

Prognostic factor and independent prognostic factor refer to univariate and multivariate results respectively. Hazard ratio and 95% CI was reported only for independent prognostic factors. CI, confidence interval.

### mRNA-protein correlation

Genes resulted as independent predictors were correlated with the expression of YAP1 and YAP1pS127 proteins. YAP1 and YAP1pS127 expression levels were always highly correlated, whereas a significant correlation between mRNA levels of Hippo genes and at least one of YAP1 or YAP1pS127 proteins was found in 7 datasets. Further details were reported in Table 3 and Supplementary Figure S2.

**Table 3:** TCGA data analyses summary.

Data set	Independent predictor (mRNA)	Worse prognosis (predictor)	Theoretical effect on Hippo pathway	Theoretical effect on TEAD-mediated transcription	Concordance with role in Hippo pathway	Correlation with YAP1 protein
OV	*MAP4K2*	high	activation	inhibition	no	no
KIRC	*TAOK3*	low	activation	inhibition	no	inverse
	*TEAD3*	high	/	activation	yes	direct
	WWTR1	low	/	activation	no	no
HNSC	*RASSF1*	low	activation	inhibition	yes	inverse
LUSC	*MAP4K2*	high	activation	inhibition	no	no
	*MINK1*	high	activation	inhibition	no	no
SKCM	PTPN14	high	activation	inhibition	no	no
	*TEAD4*	high	/	activation	yes	no
LUAD	*FRMD6*	high	activation	inhibition	no	no
BLCA	*TEAD4*	high	/	activation	yes	direct
GBM	*TEAD2*	low	/	activation	no	direct (only with YAPpS127)
	*TNIK*	low	activation	inhibition	yes	no
LGG	*TEAD2*	high	/	activation	yes	direct
LIHC	/					
CESC	*LATS2*	high	activation	inhibition	no	direct
	*MAP4K1*	low	activation	inhibition	no	no
MESO	*MAP4K4*	high	activation	inhibition	no	no
	*SAV1*	low	activation	inhibition	yes	no
PAAD	*TEAD4*	high	/	activation	yes	no

For each dataset, independent predictors, correlation with YAP1 protein and congruence with the theoretical role within Hippo pathway are indicated.

### Review of literature

**Figure 3:**
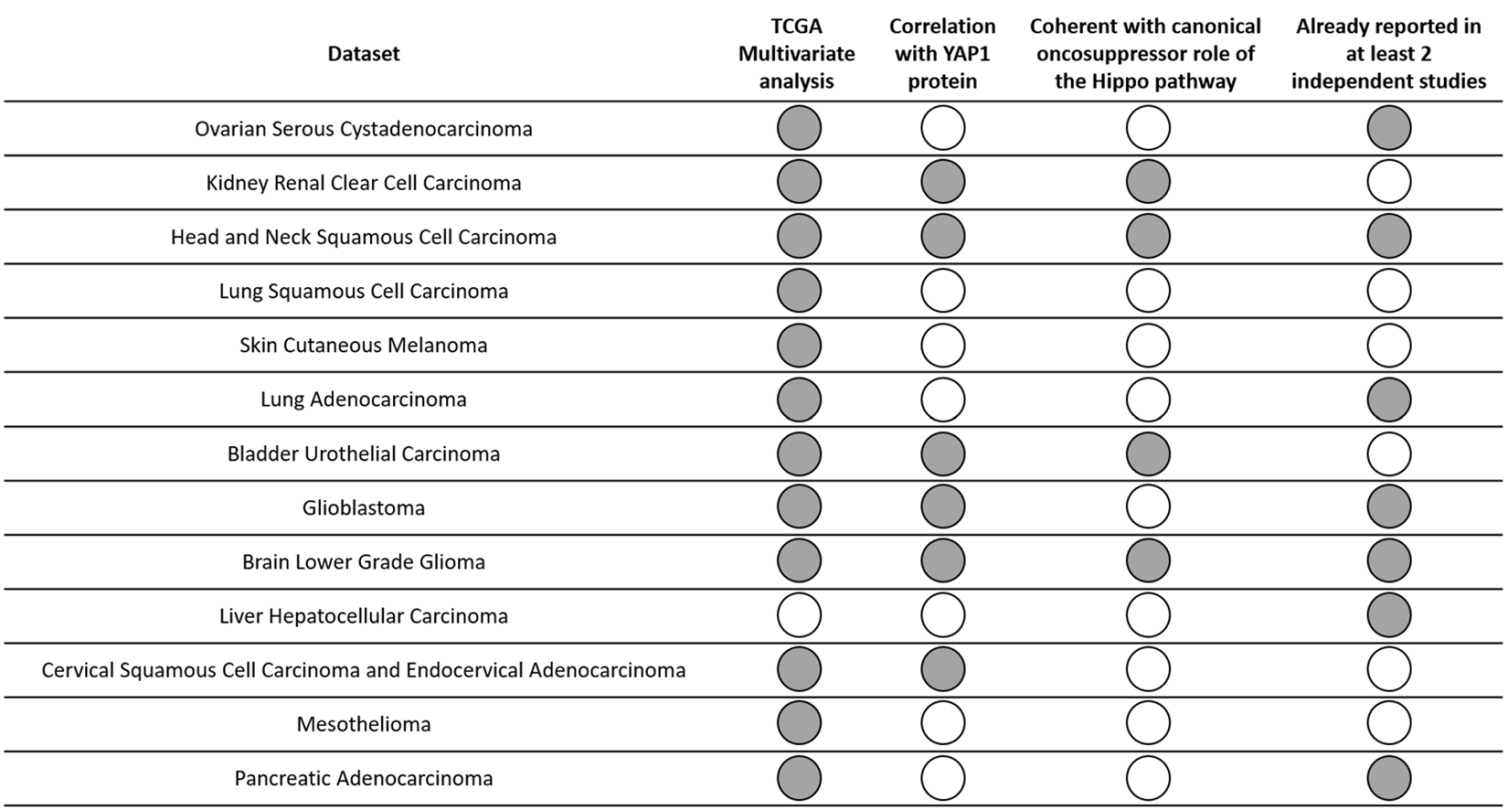
Results summary. For each analysed TCGA datasets, grey circles indicate the presence of: an independent predictor among Hippo components (multivariate survival analysis); a correlation of the independent predictor with YAP1 protein; coherence between poor survival and canonical oncosuppressor role of the Hippo pathway; and the presence of at least 2 independent studies confirming our results.

Seventy-two original articles associated 17 of the 32 Hippo genes with patients’ survival in more than 20 human cancers. Gastric and colorectal cancers were the most frequently tumors reporting association of Hippo genes with patients’ prognosis; whereas the most represented gene was *YAP1*, reported as prognostic factor in 29 different studies in 14 cancer models. The majority of these 29 studies were conducted on a protein level and, in all but 2, patients with a high expression level of YAP1 had a lower survival rate. In addition, more than 10 studies associated only nuclear and not cytoplasm staining with patients’ prognosis. Table 4 summarizes the review of literature, and Fig. 3 sums up the overall results.

**Table 4:** Review of literature.

Gene	Cancer model	Study	mRNA/ protein	n of cases	Univariate p value	Multivariate p value	worse prognosis (low/high)	Notes, score and cutoff
*LATS1*	gastric cancer	Zhang J *et al*.^17^	protein	89	0.0013	0.017	low	SE × I, max 12 (0–3 vs 4–12)
	glioma	Ji T *et al*.^18^	protein	103	<0.001	<0.001	low	SE + I, max 7 (0–1 vs 2–3 vs 4–5 vs 6–7)
	non-small-cell lung cancer	Lin X-Y *et al*.^19^	protein	136	0.035	NA	low	SE × I, max 12 (0 vs 1–3 vs 4–12)
	ovarian serous carcinoma	Xu B *et al*.^20^	protein	57	0.015	0.006	low	SE × I, max 12 (0–1 vs 4–12)
*LATS2*	nasopharyngeal carcinoma	Zhang Y *et al*.^21^	protein	220	0.007	0.037	high	SE + I, max 7, median value as cutoff
	lung adenocarcinoma	Luo SY *et al*.^22^	protein	49	0.055	0.036	low	SEP × I, max 300, mean value as cutoff
	non-small-cell lung cancer	Wu A *et al*.^23^	protein	73	0.001	0.002	low	sum of cytoplasm and nuclear staining score, the first is SE × I (0–9), the second is based on I (0–3), max 12 (0–6 vs 7–12)
*MAP4K4*	breast cancer	Zhang X *et al*.^24^	protein	82	0.021	NA	high	SE + I, max 7 (0–2 vs 3–7)
	colorectal cancer	Hao J-M *et al*.^25^	protein	181	0.029	NA	high	SE × I, max 12 (0–3 vs 4–12)
	hepatocellular carcinoma	Liu A-W *et al*.^26^	protein	400	0.019	0.014	high	median SEP as cutoff
	lung adenocarcinoma	Qiu M-H *et al*.^27^	protein	309	0.014	0.009	high	median SEP as cutoff
	pancreatic ductal adenocarcinoma	Liang JJ *et al*.^28^	protein	66	0.025	0.025	high	median SEP as cutoff
*MAP4K5*	pancreatic cancer	Wang OH *et al*.^29^	protein	105	0.02	0.012	low	negative or weak staining vs moderate or strong staining
*MOB1A*	intrahepatic cholangiocarcinoma	Sugimachi K *et al*.^30^	protein	88	0.0202	n.s.	low	SE × I, max 12, unspecified cutoff
*NF2*	hepatocellular carcinoma	Luo Z L *et al*.^31^	protein	148	0.013	NA	low	SE × I, max 12, median as cutoff
	mesothelioma	Meerang M *et al*.^32^	protein	145	0.03	0.01	low	SE × I, max 3 ( ≤ 0.5 vs > 0.5)
*RASSF1*	renal clear-cell carcinoma	Klacz J *et al*.^33^	mRNA	86	0.004	0.02	low	qRT-PCR, RASSF1A isoform, median as cutoff
	esophageal squamous cell carcinoma	Guo W *et al*.^34^	protein	141	<0.05	0.04	low	RASSF1A isoform,SE + I, max 6 (0–2 vs 3–6)
	esophageal squamous cell carcinoma	Zhang Y *et al*.^35^	protein	76	<0.001	<0.001	low	SE + I, max 6 (0–1 vs 2–6)
*RASSF6*	colorectal cancer	Zhou R *et al*.^36^	protein	127	<0.001	0.03	low	SE × I, ROC curve to set the cutoff
	gastric cancer	Wen Y *et al*.^37^	protein	264	<0.001	<0.001	low	SE + I, max 6 (0–2 vs 3–4 vs 5–6)
	gastric cardia adenocarcinoma	Guo W *et al*.^38^	protein	106	<0.05	0.04	low	SE + I, max 6 (0–2 vs 3–6)
	pancreatic ductal adenocarcinoma	Ye H-L *et al*.^39^	protein	96	0.021	0.006	low	SE + I, max 6 (0–2 vs 3–6)
*SAV1*	pancreatic ductal adenocarcinoma	Wang L *et al*.^40^	protein	83	<0.001	0.002	low	SE × I, max 9 (0–3 vs 4–9)
*STK4*	breast cancer	Lin X *et al*.^41^	protein	110	0.027	0.03	low	10% of SEP as cutoff
	breast cancer	Lin X-Y *et al*.^42^	protein	98	0.010	0.002	low	detection on plasma by ELISA, average as cutoff
	colorectal cancer	Yu J *et al*.^43^	mRNA	46	0.0008	NA	low	microarray, ROC curve to set the cutoff
	colorectal cancer	Minoo P *et al*.^44^	protein	1420	0.014 0.0001	n.s. 0.03	low	SEP, ROC curve to set the cutoff, p values refer to mismatch-repair proficient and deficient subgroups respectively
	colorectal cancer	Zlobec I *et al*.^45^	protein	1420	0.002	<0.05	low	SEP, ROC curve to set the cutoff
*TEAD1*	hepatocellular carcinoma	Ge X and Gong L 2017^46^	mRNA	60	0.002	NA	high	qRT-PCR, relative log2 transformation (positive vs negative log2 values)
	prostate cancer	Knight JF 2008^47^	protein	147	0.0092 0.0009	n.s. 0.037	high high	p values refer to SE (zero vs focal vs diffuse) and I (0 vs 1 vs 2 vs 3) respectively, considered as separate parameters
*TEAD4*	colorectal cancer	Liu Y *et al*.^48^	protein	416	0.0002	NA	high	nuclear staining, positive vs negative staining
	ovarian cancer	Xia Y *et al*.^49^	protein	45	<0.001	NA	high	SE + I, max 5 (0–1 vs 2–5)
*TNIK*	colorectal cancer	Takahashi H *et al*.^50^	protein	220	<0.001	0.011	high	expression of the protein at the invasive tumor front, SE + I, max 7 (0–5 vs 6–7)
	hepatocellular carcinoma	Jin J *et al*.^51^	protein	302	0.001	0.003	high	phosphorylated protein, negative or weak vs moderate or strong
	pancreatic cancer	Zhang Y *et al*.^52^	protein	91	0.021	n.s.	high	SEP, median value as cutoff
*VGLL4*	gastric cancer	Jiao S *et al*.^53^	protein	91	0.0416	0.0215	low	SE × I, max 12 (0–1 vs 2–12)
*WWC1*	gastric cancer	Yoshihama Y *et al*.^54^	protein	164	0.037	NA	high	low expression of atypical protein kinase Cλ/τ subgroup, I compared to normal tissue, score 2 is comparable to normal tissue staining, max 3 (0–1 vs 2–3)
*WWTR1*	colorectal cancer	Wang L *et al*.^55^	protein	168	<0.001	0.050	high	SE × I, max 12 (0–4 vs 5–12)
	esophagogastric junction adenocarcinoma	Sun L *et al*.^56^	protein	135	<0.001	0.022	high	SE × I, max 12 (0–4 vs 5–12)
	hepatocellular carcinoma	Guo Y *et al*.^57^	protein	180	<0.05	NA	high	SE × I, max 12 (0–4 vs 5–12)
	hepatocellular carcinoma	Hayashi H *et al*.^58^	mRNA	110	<0.05	NA	high	qRT-PCR, 70th percentile as cutoff
	non-small-cell lung cancer	Xie M *et al*.^59^	protein	181	0.002	0.006	high	positive vs negative staining
	oral cancer	Li Z *et al*.^60^	protein	111	0.0008	0.003	high	SE × I, max 12 (0–4 vs 5–12)
	retinoblastoma	Zhang Y *et al*.^61^	protein	43	0.048	0.049	high	unspecified cutoff
	tongue squamous cell carcinoma	Wei Z *et al*.^62^	protein	52	0.0204	0.008	high	SE × I, max 12 (0–4 vs 5–12)
	uterine endometrioid adenocarcinoma	Zhan M *et al*.^63^	protein	55	0.018	n.s.	high	SEP × I, max 300 (<100 vs >100)
*YAP1*	adrenocortical cancer	Abduch R H *et al*.^64^	mRNA	31	0.05	NA	high	pediatric patients, qRT-PCR, unspecified cutoff
	bladder urothelial carcinoma	Liu J-Y *et al*.^65^	protein	213	<0.001	0.003	high	positive vs negative staining
	breast cancer	Cao L *et al*.^66^	protein	324	0.005	NA	low	nuclear staining, SEP × I, max 300, median value as cutoff, luminal A subgroups
	breast cancer	Kim H M *et al*.^67^	protein	122	0.008 0.003	NA	high high	metastatic patients, nuclear staining, SE × I, max 6 (0–1 vs 2–6), p values refer to YAP e pYAP respectively
	breast cancer	Kim S K *et al*.^68^	protein	678	0.024	n.s.	high	nuclear staining, negative or weak staining vs moderate or strong staining in more than 10% of tumor area
	intrahepatic cholangiocarcinoma	Sugimachi K *et al*.^30^	protein	88	0.0242	0.0093	high	nuclear staining, SE × I, max 12 (0–3 vs 4–12)
	cholangiocarcinoma	Pei T *et al*.^69^	protein	90	0.016	0.026	high	negative or weak vs strong staining, the cutoff between weak and strong staining is the median value of the integrated optical density
	colorectal cancer	Wang Y *et al*.^70^	protein	139	0.0003	0.0207	high	positive vs negative staining, positive defined as strong cytoplasmic staining in more than 50% of tumor cells or nuclear staining in more than 10% of tumor cells
	colorectal cancer	Wang L *et al*.^55^	protein	168	0.006	0.021	high	SE × I, max 12 (0–4 vs 5–12)
	esophageal squamous cell carcinoma	Yeo M-K *et al*.^71^	protein	142	0.006	0.034	high	nuclear staining, SE × I, mean value as cutoff
	gallbladder cancer	Li M *et al*.^72^	protein	52	<0.01	0.020	high	nuclear staining, SE + I, max 6 (0–2 vs 3–6)
	gastric cancer	Huang S *et al*.^73^	protein	120	<0.001	<0.001	high	nuclear staining, SE × I, max 9 (0–3 vs 4–9)
	gastric cancer	Sun D *et al*.^74^	protein	270	<0.001	NA	high	SE × I, max 12 (0–3 vs 4–12)
	gastric adenocarcinoma	Li P *et al*.^75^	protein	161	0.001	0.015	high	SE × I, max 12 (0–3 vs 4–12)
	intestinal type gastric cancer	Song M *et al*.^76^	protein	117	0.024	0.018	high	nuclear staining, SEP (50% as cutoff)
	gastric cancer	Kang W *et al*.^77^	protein	129	0.021	n.s.	high	nuclear staining, SEP (0% vs ≤ 10% vs 10% to 50% vs > 50%), YAP1 nuclear staining is an independent prognostic marker in stage I-II subgroup
	glioma	Liu M *et al*.^78^	protein	72	0.0002	<0.001	high	staining quantified by software
	cholangiocarcinoma	Lee K *et al*.^79^	protein	88	0.005	NA	high	intrahepatic pT1 subgroup, nuclear staining, staining intensity ≥ 2 + in more than 5% of tumor cells as cutoff
	hepatocellular carcinoma and hepatic cholangiocarcinoma	Wu H *et al*.^80^	protein	137 122	0.001 0.013	0.008 0.026	high high	SE × I, max 12 (0–3 vs 4–12)
	hepatocellular carcinoma	Xu B *et al*.^81^	protein	89	<0.001	NA	high	unspecified cutoff
	hepatocellular carcinoma	Hayashi H *et al*.^58^	mRNA	110	<0.05	NA	high	qRT-PCR, 75th percentile as cutoff
	hepatocellular carcinoma	Han S-X *et al*.^82^	protein	39	0.042	0.005	high	SE × I, max 12 (0–3 vs 4–12)
	lung adenocarcinoma	Sun P-L *et al*.^83^	protein	205	0.001	0.013	low	cytoplasmic staining, strong cytoplasmic staining in more than 50% of tumor cells as cutoff
	melanoma	Menzel M *et al*.^84^	protein	380	0.013	NA	high	staining compared to that of hair bulb stem cells: 0 = no staining, 1 = weaker, 2 = comparable, 3 = stronger (0 vs 1 vs 2 vs 3)
	ovarian cancer	He C *et al*.^85^	protein	342	0.018	NA	high	staining quantified by software
	ovarian cancer	Xia Y *et al*.^49^	protein	46	0.002	NA	high	SE × I, max 5 (0–1 vs 2–5)
	pancreatic ductal adenocarcinoma	Salcedo Allende MT *et al*.^86^	protein	64	0.072	0.032	high	SEP × I, max 300, unspecified cutoff
	pancreatic ductal adenocarcinoma	Zhao X *et al*.^87^	protein	96	<0.001	0.004	high	SE × I, max 9 (0–4 vs 5–9)
	pancreatic ductal adenocarcinoma	Wei H *et al*.^88^	protein	63	<0.05	NA	high	nuclear staining,SEP, 10% as cutoff

In univariate and multivariate p values columns, p are reported as indicated in the study. SE staining extend; I intensity; SEP staining extend percentage; NA not available; n.s. not significant.

## Discussion

Genetic alterations affecting the Hippo pathway components are generally rare events in the cancer biology landscape, except for malignant pleural mesothelioma and some tumors of the nervous system, such as neurofibromas, meningiomas and shwannomas^4,10,11^. However, the disruption of this pathway was reported in several human cancers. Epigenetic events, post-transcriptional and post-translational modifications can all play a crucial effect on this pathway^12^, and simultaneously monitoring all these alterations is impracticable. If a positive aspect can exist in this scenario, it is the converging effect of a great variety of dysregulation on a single protein expression and/or phosphorylation, YAP1. Herein, we investigated the effect of mRNA and protein levels of the Hippo pathway components on survival of cancer patients by both analysing TCGA data and reviewing the literature.

In the large majority of analysed datasets, the mRNA levels of the Hippo pathway components were associated with patients’ survival, and most importantly, in almost all cancer models taken into account at least one of the considered genes was an independent predictor (Table 2). We then decided to move another step forward, on a protein level, to understand if the predictors correlated with the effector, YAP1 protein and its phosphorylation status.

The protein levels from TCGA were obtained by standard reverse phase protein lysate microarray, a technique that allows to reliably estimate protein levels and post-translational modifications, without considering the initial compartmentalization^13^. As a consequence, we always found a very high direct correlation between YAP1 and YAP1pS127 that theoretically should determine a very different output: TEAD-mediated transcription and YAP1 inactivation respectively. Considering that this incongruence should be overcome by other techniques such as immunohistochemistry (IHC), we found that 7 of the 19 predictors were correlated with high levels of YAP1 protein (Table 3). Interestingly, *MAP4Ks* never correlated with YAP1 protein, and, when they were independent predictors, very often the expression levels associated with a worse prognosis were not justified by their theoretical role within Hippo pathway. Nevertheless, this is in agreement with other well-known functions of MAP4Ks^14^ and with 8 out of 9 previous studies that associated high MAP4Ks levels with a worse prognosis (Table 4). Assuming that MAP4Ks should not play a pivotal role in the regulation of Hippo pathway, more than half (7 out of 12) of the other independent predictors were correlated with YAP1. In addition, because of mRNA levels were compared with survival of patients, some incongruence should be accounted for feedback mechanisms such as in the case of *LATS2*. In fact, *LATS2* is a direct transcriptional target of activated YAP1-WWTR1-TEADs^15^, thus explaining high *LATS2* mRNA levels associated with poor prognosis.

Yet, more than half of Hippo genes were already associated with patients’ prognosis in different independent studies in several human cancers (Table 4). High expression levels of YAP1 were repeatedly reported as a poor prognostic factor, especially in gastric, colorectal, hepatocellular, pancreatic and lung cancer. These cancer types should then really benefit from treatment with YAP1 inhibitors, as well as kidney renal clear cell carcinoma, head and neck carcinoma, bladder cancer and lower grade glioma, in which we found not only at least one Hippo gene as an independent prognostic factor, but also a correlation between the predictors and YAP1 protein levels, coherently with their role within Hippo pathway.

In conclusion, the independent impact of YAP1 activation on patients’ survival was repeatedly proven by several independent studies and in a large variety of human cancers. Several molecules can disrupt YAP1 activation, and showed very promising results both *in vitro* and in mice. Some of these molecules directly bind to YAP1 thus allowing to use its expression levels as a potential predictive biomarker. Moreover, YAP1 evaluation by IHC would provide not only the direct quantification of the protein levels, but also the visualization of its compartmentalization: this is a relevant point because nuclear YAP1 is the real biological effector and strongly correlated with patients prognosis. Indeed, YAP1 quantification by IHC needs to be uniformly assessed because of the wide interpretation criteria that still exist.

Finally, Kary Mullis truly said that the majority of the scientific studies are correlation and not cause-effect, but when a great number of independent studies point in the same direction, maybe the time is ripe to move a step forward.

## Methods

### Selection of genes and datasets

Thirty-two genes belonging to the core Hippo pathway were considered in the present study (Table 5). Level 3 RNA Seq, level 3 reverse phase protein lysate microarray and clinical data of all solid tumor datasets of TCGA except pure sarcomas were downloaded from cBioPortal (www.cbioportal.org). In order to select datasets for further investigation, power analysis for survival data was performed with the powerSurvEpi R package version 0.0.9. In detail, two hypothetical groups with the same number of patients and the same probability of death were considered. Moreover, postulated risk ratio of 2.3 and alpha of 0.05 were set to assess the statistical power of each dataset. Datasets with β above 0.8 were selected for further analyses.

**Table 5:** List of Hippo genes considered in the study.

Gene	Entrez gene id	Approved name
*FRMD6*	122786	FERM domain containing 6
*LATS1*	9113	large tumor suppressor kinase 1
*LATS2*	26524	large tumor suppressor kinase 2
*MAP4K1*	11184	mitogen-activated protein kinase kinase kinase kinase 1
*MAP4K2*	5871	mitogen-activated protein kinase kinase kinase kinase 2
*MAP4K3*	8491	mitogen-activated protein kinase kinase kinase kinase 3
*MAP4K4*	9448	mitogen-activated protein kinase kinase kinase kinase 4
*MAP4K5*	11183	mitogen-activated protein kinase kinase kinase kinase 5
*MINK1*	50488	misshapen like kinase 1
*MOB1A*	55233	MOB kinase activator 1A
*MOB1B*	92597	MOB kinase activator 1B
*NF2*	4771	neurofibromin 2
*PTPN14*	5784	protein tyrosine phosphatase, non-receptor type 14
*RASSF1*	11186	Ras association domain family member 1
*RASSF6*	166824	Ras association domain family member 6
*SAV1*	60485	salvador family WW domain containing protein 1
*STK3*	6788	serine/threonine kinase 3
*STK38*	11329	serine/threonine kinase 38
*STK38L*	23012	serine/threonine kinase 38 like
*STK4*	6789	serine/threonine kinase 4
*TAOK1*	57551	TAO kinase 1
*TAOK2*	9344	TAO kinase 2
*TAOK3*	51347	TAO kinase 3
*TEAD1*	7003	TEA domain transcription factor 1
*TEAD2*	8463	TEA domain transcription factor 2
*TEAD3*	7005	TEA domain transcription factor 3
*TEAD4*	7004	TEA domain transcription factor 4
*TNIK*	23043	TRAF2 and NCK interacting kinase
*VGLL4*	9686	vestigial like family member 4
*WWC1*	23286	WW and C2 domain containing 1
*WWTR1*	25937	WW domain containing transcription regulator 1
*YAP1*	10413	Yes associated protein 1

### Survival and correlation analyses

For each dataset, clinical-pathological features mainly affecting patients’ survival according to the eighth edition of the American Joint Committee on Cancer^16^ were taken into account as covariates. In order to directly compare the effect of genes and covariates, patients with missing values for any of the selected clinical-pathological parameters were removed from the analyses. For each gene, patients were divided into two groups, high and low expression levels, based on the median value. Also for age, the median was used to dichotomize patients. Survival curves were estimated with the Kaplan-Meier method and compared using the log-rank test. Multivariate Cox proportional hazard modelling of genes and covariates identified as potential prognostic factors in the univariate analyses was then used to determine their independent impact on patients’ survival, and to estimate the corresponding hazard ratio, setting high expression as reference group. All survival analyses were performed with the survival R package version 2.41-3. All p values below 0.05 were considered to be statistically significant.

**Figure 4:**
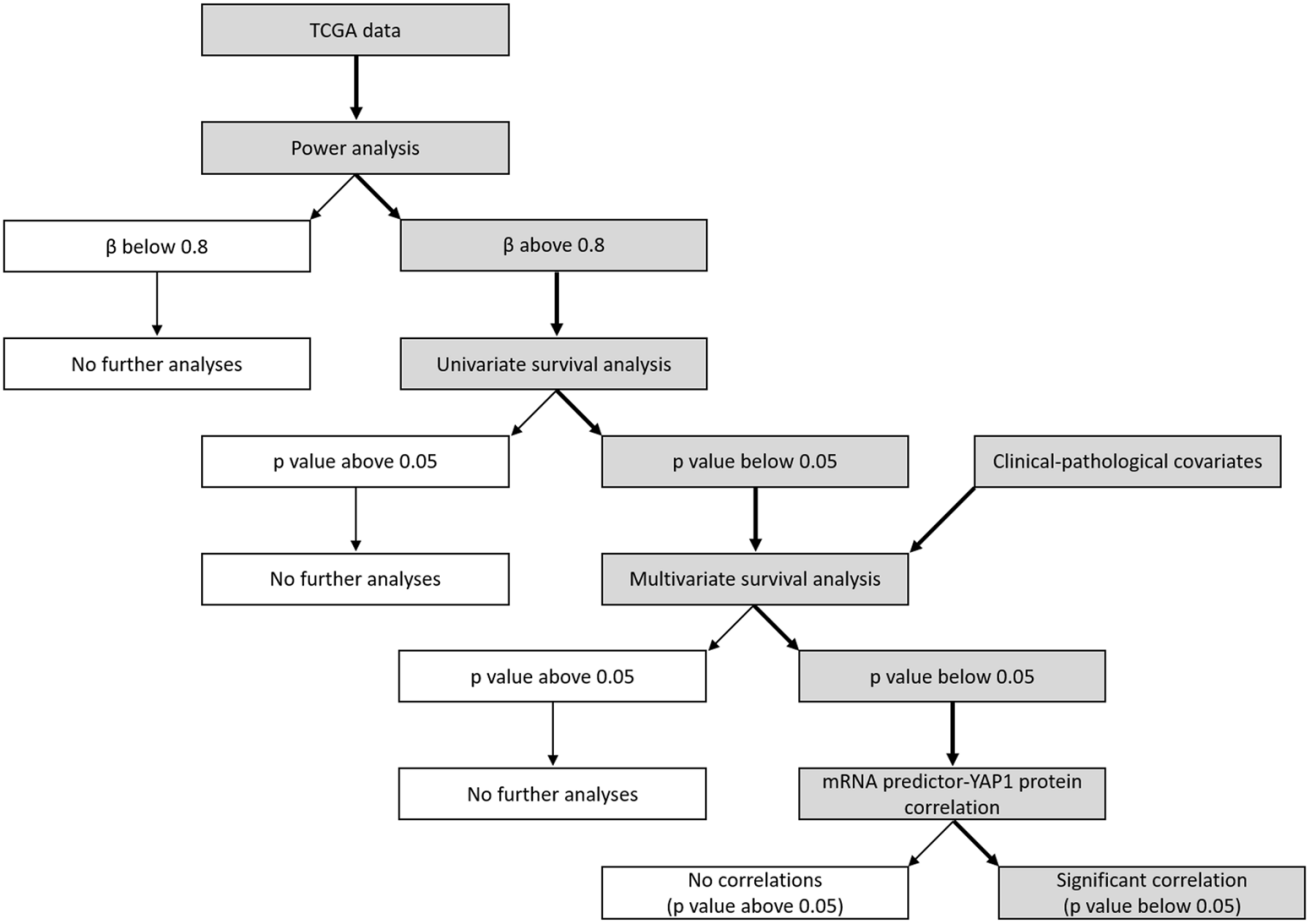
Flow chart of data analyses. Bold arrows and grey rectangles highlight the main path that led to obtained results and conclusions.

All genes identified as independent prognostic factors were correlated with YAP1 and YAP1pS127 protein expression levels using Pearson’s correlation, following the procedures of Hmisc R package version 4.1-1. The flow chart of data analyses is reported in Fig. 4.

### Review of literature

PubMed database (www.ncbi.nlm.nih.gov/pubmed) was used to search papers investigating Hippo genes and survival of cancer patients. All aliases provided by HUGO nomenclature (www.genenames.org) were used. Only English-written original articles were selected, and only papers containing original data and concerning protein or mRNA levels were considered.

### Data availability

The datasets analysed during the current study are available at www.cbioportal.org.

## Electronic supplementary material

Supplementary Information
