# Supplementary material for: Hippo pathway affects survival of cancer patients: extensive analysis of TCGA data and review of literature
Source: Sci Rep. 2018 Jul 13;8:10623. doi: 10.1038/s41598-018-28928-3 (PMC6045671; doi:10.1038/s41598-018-28928-3)

Hippo pathway affects survival of cancer patients: extensive analysis of TCGA data and review of literature

Anello Marcello Poma[1], Liborio Torregrossa[2], Rossella Bruno[1], Fulvio Basolo*[1] and Gabriella Fontanini[1]

[1]Department of Surgical, Medical, Molecular Pathology and Critical Area, University of Pisa, Pisa, Italy

[2]Department of Laboratory Medicine, Section of Pathology, University Hospital of Pisa, Pisa, Italy

Supplementary table S1. p values of univariate (log-rank test) survival analysis of Hippo genes and selected clinical-pathological parameters.

Variable	OV	KIRC	HNSC	LUSC	SKCM	LUAD	BLCA	GBM	LGG	LIHC	CESC	MESO	PAAD
FRMD6	0.8048	0.0088	0.1762	0.2307	0.2967	0.0040	0.8030	0.1318	0.1618	0.7139	0.2655	0.0285	0.0419
LATS1	0.5145	0.0387	0.8844	0.3155	0.9583	0.1674	0.3603	0.6771	0.7205	0.8433	0.0360	0.4416	0.5438
LATS2	0.0694	0.0055	0.4382	0.0176	0.6041	0.0064	0.3307	0.4719	0.0000	0.7619	0.0000	0.4920	0.4907
MAP4K1	0.2247	0.0015	0.0297	0.2629	0.3345	0.0847	0.0584	0.2691	0.0002	0.4696	0.0040	0.9579	0.2043
MAP4K2	0.0222	0.5609	0.3991	0.0029	0.8323	0.1002	0.1379	0.0411	0.0559	0.0661	0.8132	0.2060	0.1268
MAP4K3	0.2779	0.6650	0.3923	0.2671	0.2775	0.8621	0.1343	0.1016	0.5225	0.8834	0.1288	0.8954	0.0697
MAP4K4	0.3371	0.5305	0.1447	0.0820	0.9202	0.0617	0.7934	0.5739	0.0882	0.1278	0.0649	0.0007	0.0055
MAP4K5	0.2399	0.6646	0.3674	0.0451	0.5044	0.3031	0.1831	0.2515	0.0538	0.4274	0.2914	0.4462	0.0695
MINK1	0.9732	0.1454	0.3296	0.0175	0.2381	0.8170	0.7357	0.6473	0.7446	0.2425	0.7556	0.8406	0.1012
MOB1A	0.0946	0.1173	0.0602	0.4365	0.2471	0.3165	0.4794	0.5017	0.0009	0.2456	0.2778	0.4163	0.0376
MOB1B	0.1457	0.8443	0.7680	0.4908	0.1227	0.7926	0.6045	0.4771	0.0009	0.4506	0.4124	0.1270	0.2572
NF2	0.8677	0.2249	0.8404	0.3917	0.0655	0.3026	0.3421	0.7721	0.0477	0.2452	0.1407	0.4800	0.0217
PTPN14	0.9361	0.0145	0.4932	0.5944	0.0323	0.3230	0.1330	0.9865	0.1574	0.3741	0.0934	0.2066	0.0003
RASSF1	0.2042	0.0228	0.0020	0.5315	0.9772	0.5651	0.2201	0.0132	0.0448	0.7485	0.1690	0.0720	0.3789
RASSF6	0.3289	0.0358	0.4755	0.2996	0.2477	0.9872	0.8021	0.2107	0.3991	0.4807	0.4038	0.0429	0.8938
SAV1	0.2423	0.0011	0.6462	0.6162	0.2171	0.8833	0.2270	0.9914	0.7972	0.2824	0.2467	0.0012	0.0002
STK3	0.3556	0.8844	0.2677	0.5537	0.2637	0.0604	0.8254	0.7598	0.0001	0.8245	0.5046	0.9295	0.0042
STK38	0.4202	0.1025	0.1515	0.6628	0.8626	0.6765	0.0642	0.1447	0.0012	0.4554	0.2989	0.4008	0.2393
STK38L	0.3698	0.0957	0.5444	0.3582	0.2262	0.0799	0.3802	0.3771	0.0677	0.8309	0.6633	0.0095	0.0852
STK4	0.2783	0.1476	0.9769	0.0782	0.3090	0.3069	0.2782	0.4968	0.0007	0.5229	0.5439	0.3976	0.4988
TAOK1	0.5407	0.0062	0.5068	0.2639	0.5579	0.3510	0.4143	0.9964	0.4189	0.8948	0.1769	0.2726	0.4530
TAOK2	0.5873	0.6088	0.0414	0.9959	0.2016	0.0652	0.5858	0.8381	0.0179	0.6295	0.7897	0.0770	0.0216
TAOK3	0.2117	0.0003	0.2078	0.2351	0.0384	0.7914	0.7361	0.2648	0.2418	0.3146	0.4596	0.0336	0.0673
TEAD1	0.0675	0.0002	0.3489	0.3687	0.7872	0.2435	0.7426	0.3122	0.5988	0.2086	0.2786	0.6110	0.2996
TEAD2	0.9476	0.5341	0.8006	0.5947	0.4613	0.7336	0.8791	0.0215	0.0000	0.1183	0.2418	0.1310	0.5646
TEAD3	0.7864	0.0000	0.7729	0.1343	0.3483	0.7057	0.1115	0.3442	0.0008	0.8660	0.9253	0.8420	0.4751
TEAD4	0.1709	0.0001	0.9150	0.6371	0.0223	0.0305	0.0087	0.6248	0.0015	0.2428	0.5211	0.2239	0.0059
TNIK	0.9383	0.0002	0.9278	0.2526	0.6744	0.7155	0.6449	0.0148	0.9359	0.1376	0.0272	0.0013	0.3388
VGLL4	0.9046	0.8136	0.2432	0.1621	0.3919	0.9465	0.8686	0.5433	0.0147	0.5819	0.2421	0.0101	0.4997
WWC1	0.2084	0.1063	0.0082	0.0414	0.0568	0.7246	0.9621	0.3471	0.5718	0.8476	0.3462	0.1569	0.5686
WWTR1	0.7966	0.0000	0.9527	0.0839	0.9701	0.5590	0.2573	0.7489	0.0061	0.4035	0.8678	0.1604	0.3096
YAP1	0.4207	0.9931	0.5978	0.5228	0.8428	0.4047	0.6543	0.9249	0.0040	0.9323	0.0573	0.9318	0.0002
age	0.0489		0.1304	0.2957		0.9142	0.0345	0.0710	0.0000				
clinical stage	0.2116		0.4139								0.0001		
grade	0.0642						0.8618		0.0000	0.2519			
pathologic tumor stage		0.0000		0.1776	0.0068	0.0000	0.0000			0.0144		0.5152	0.0152
residual tumor													0.0256
tobacco smoking history indicator			0.9599										
vascular invasion										0.0103			

Supplementary table S2. p values of multivariate (Cox regression) survival analysis. Only genes and clinical-pathological features identified as potential prognostic factors in the univariate analyses were used.

Variable	OV	KIRC	HNSC	LUSC	SKCM	LUAD	BLCA	GBM	LGG	LIHC	CESC	MESO	PAAD
FRMD6		0.3692				0.0304						0.1342	0.2577
LATS1		0.0527									0.3400		
LATS2		0.5626		0.0557		0.0799			0.0830		0.0020		
MAP4K1		0.8603	0.3122						0.1139		0.0318		
MAP4K2	0.0323			0.0064				0.0550					
MAP4K3													
MAP4K4												0.0191	0.4470
MAP4K5				0.0830									
MINK1				0.0297									
MOB1A									0.2269				0.3387
MOB1B									0.6823				
NF2									0.6088				0.3229
PTPN14		0.1153			0.0240								0.1119
RASSF1		0.7981	0.0088					0.0898	0.5360				
RASSF6		0.9479										0.7062	
SAV1		0.7813										0.0066	0.3025
STK3									0.4236				0.5805
STK38									0.6498				
STK38L												0.7191	
STK4									0.2172				
TAOK1		0.7900											
TAOK2			0.1766						0.8687				0.2128
TAOK3		0.0103			0.0620							0.2050	
TEAD1		0.2758											
TEAD2								0.0067	0.0434				
TEAD3		0.0488							0.5281				
TEAD4		0.0603			0.0346	0.1746	0.0351		0.9785				0.0041
TNIK		0.4179						0.0457			0.6558	0.8255	
VGLL4									0.4501			0.4461	
WWC1				0.3646									
WWTR1		0.0201	0.4225						0.8245				
YAP1									0.9729				0.1222
age	0.0694						0.0168		0.0000				
clinical stage											0.0161		
grade									0.0009				
pathologic tumor stage		0.0000			0.1177	0.0001	0.0000			0.0067			0.1063
residual tumor													0.0009
tobacco smoking history indicator													
vascular invasion										0.0137			

Supplementary figure S1. Kaplan-Meier curves of genes and clinical-pathological features resulted as independent prognostic factors.

BLCA – Bladder Urothelial Carcinoma

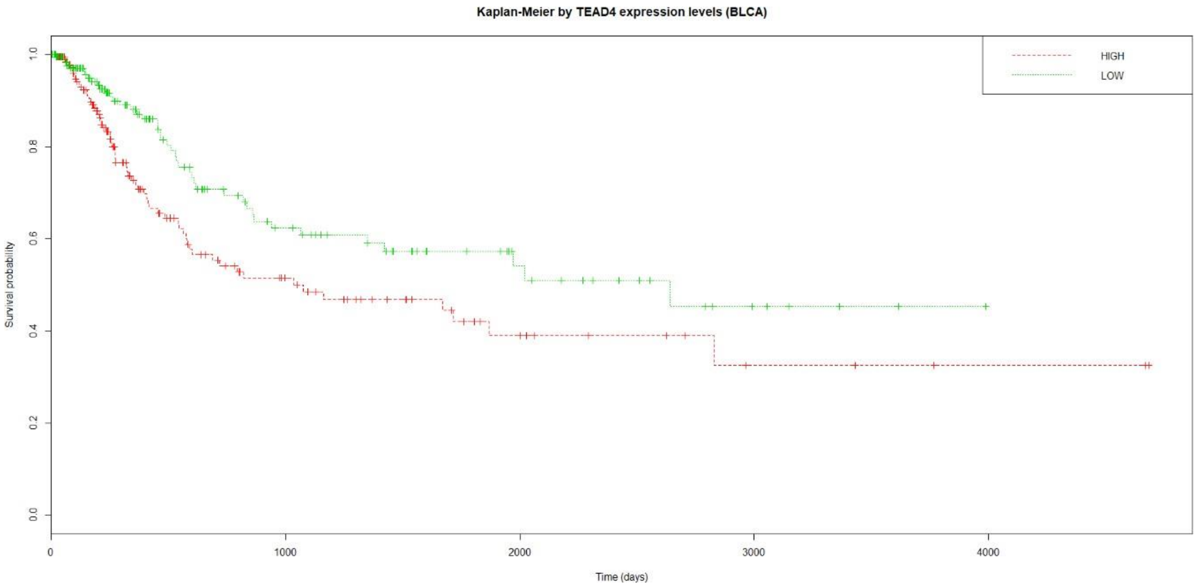

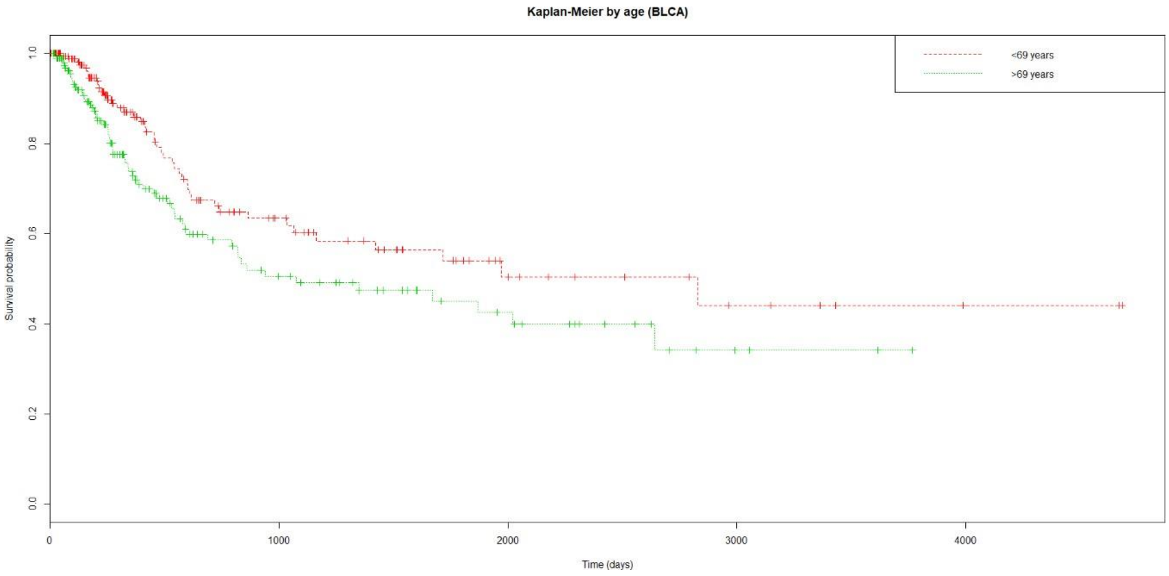

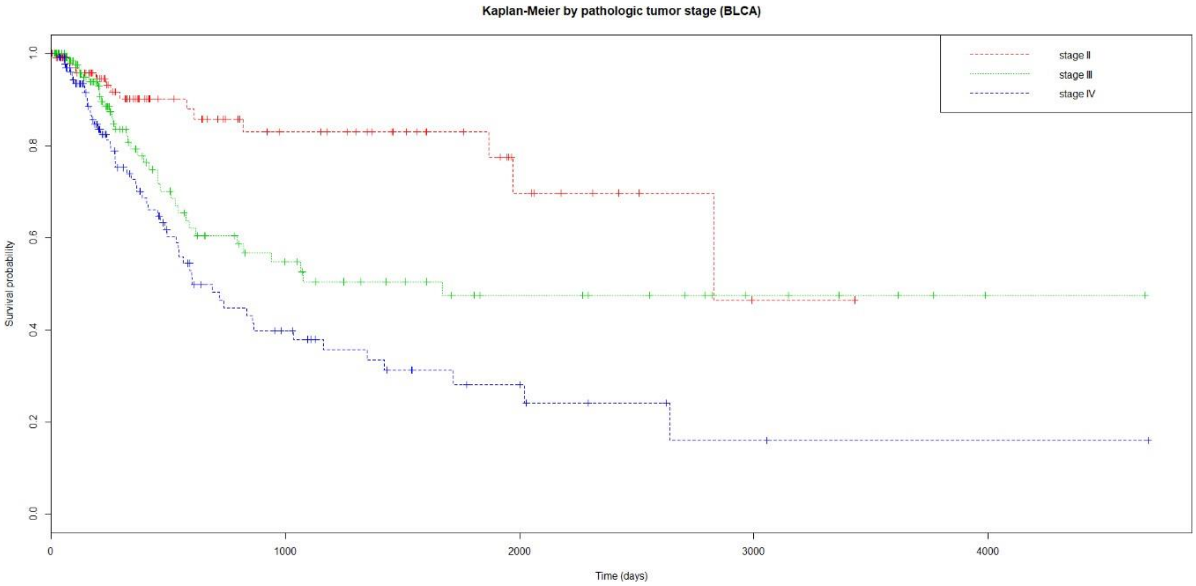

LGG – Brain Lower Grade Glioma

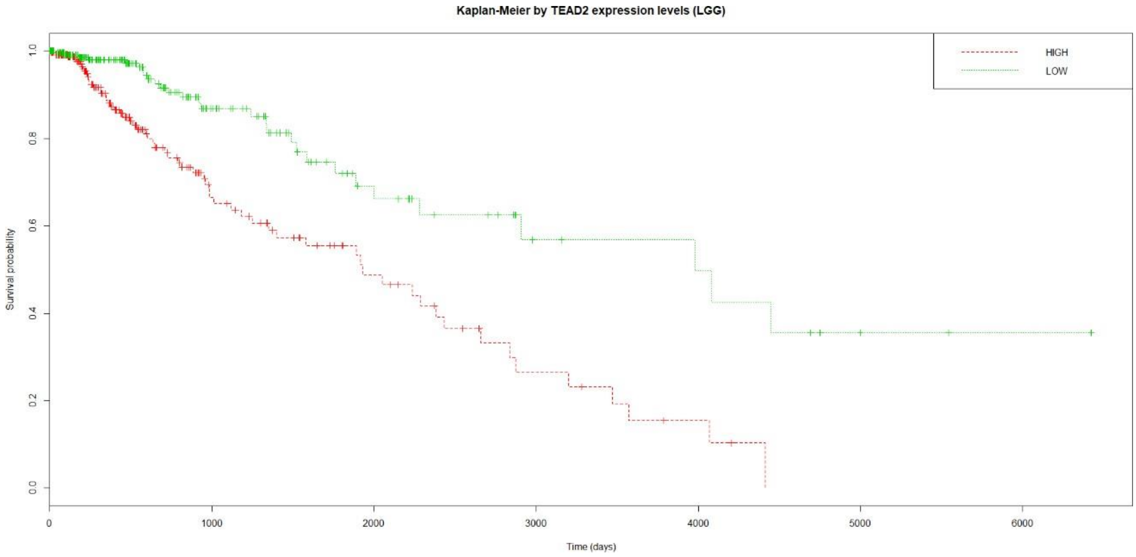

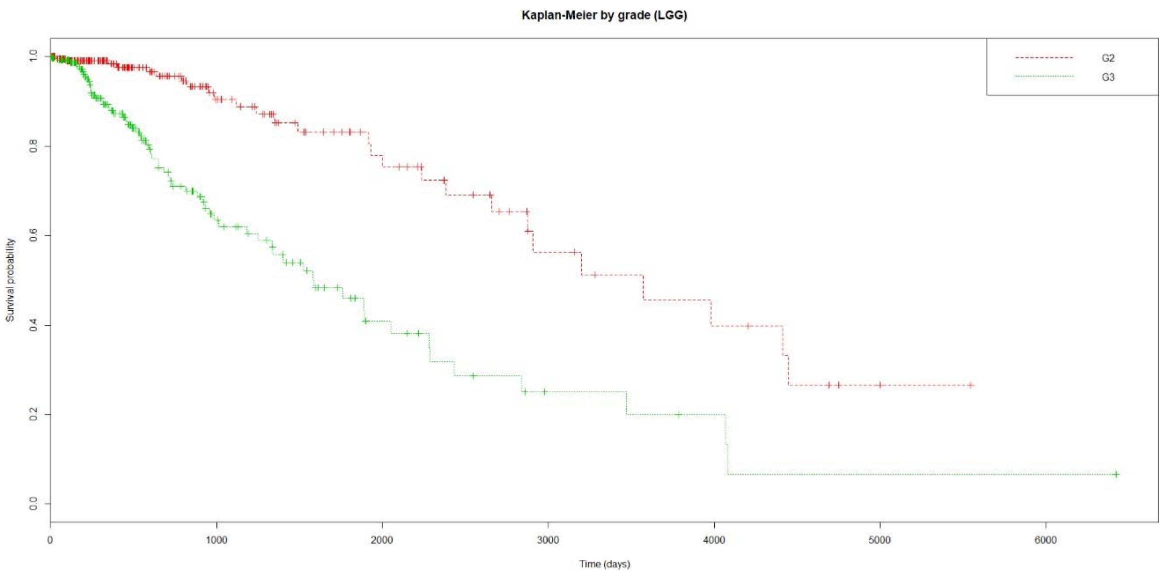
Kaplan-Meier by grade (LGG)
G2
G3
Survival probability
0.0
0.2
0.4
0.6
0.8
1.0
0
1000
2000
3000
4000
5000
6000
Time (days)

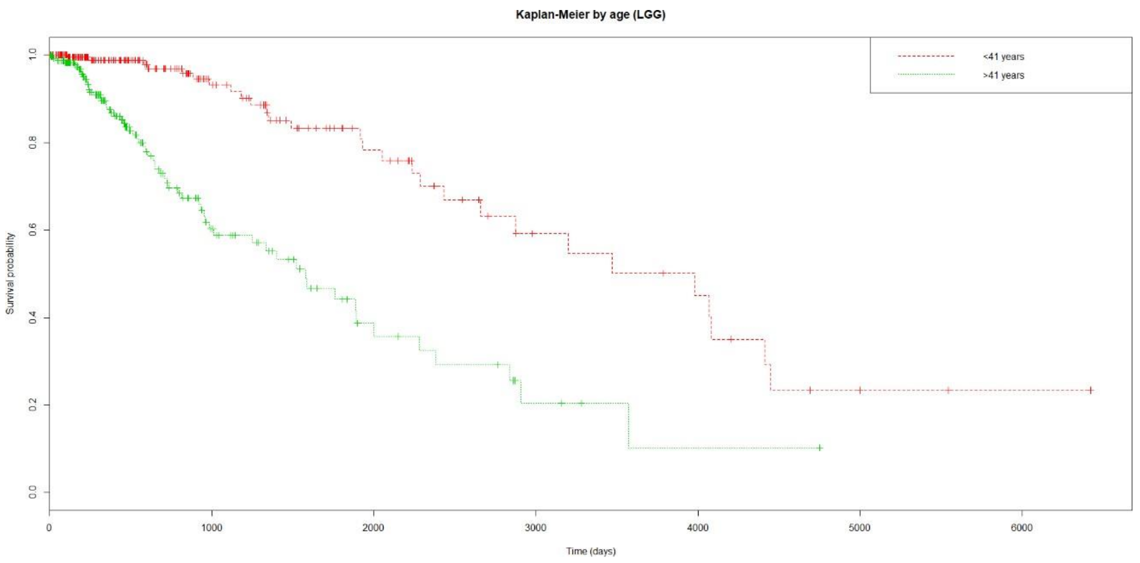
Kaplan-Meier by age (LGG)
<41 years
>41 years
Survival probability
0.0
0.2
0.4
0.6
0.8
1.0
0
1000
2000
3000
4000
5000
6000
Time (days)

CESC – Cervical Squamous Cell Carcinoma and Endocervical Adenocarcinoma

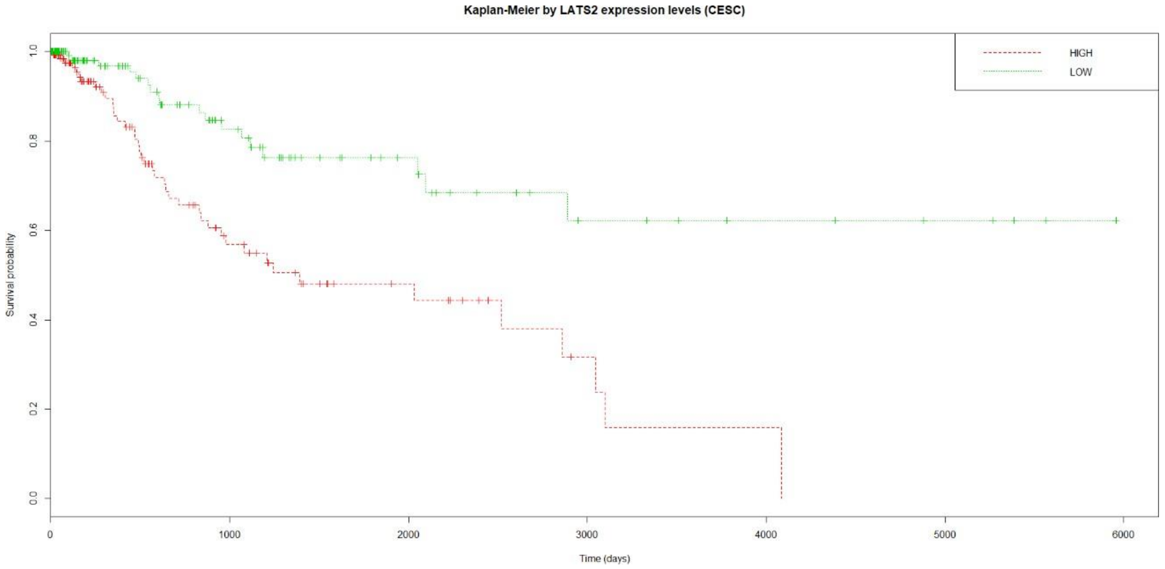

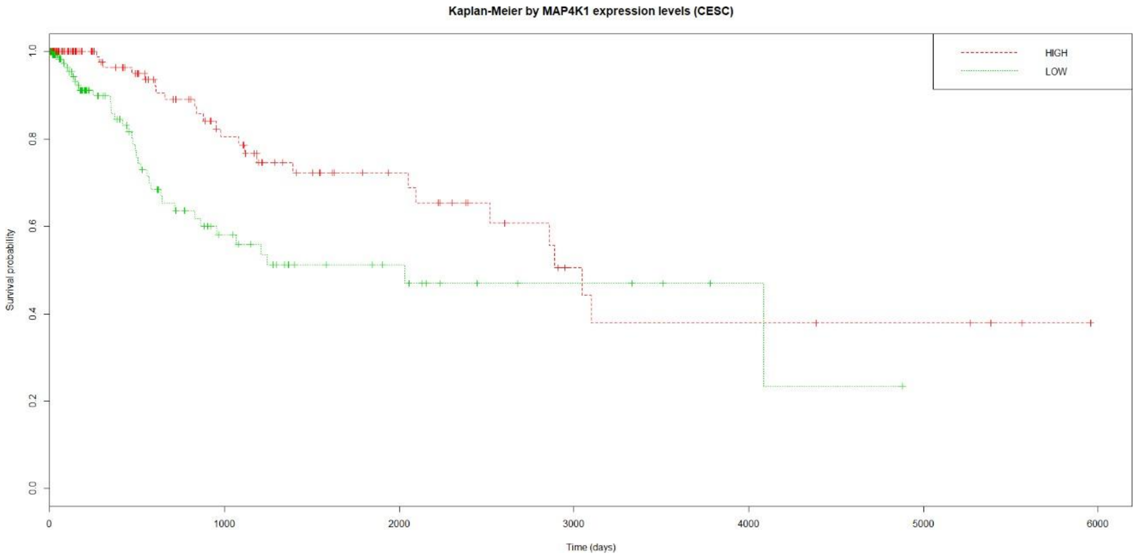

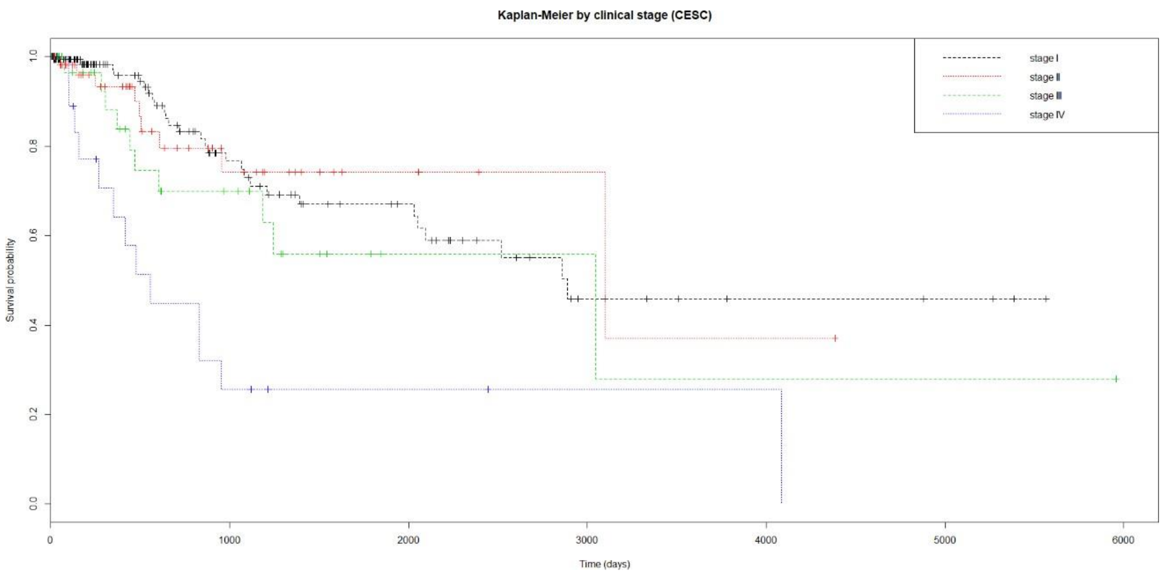

GBM – Glioblastoma

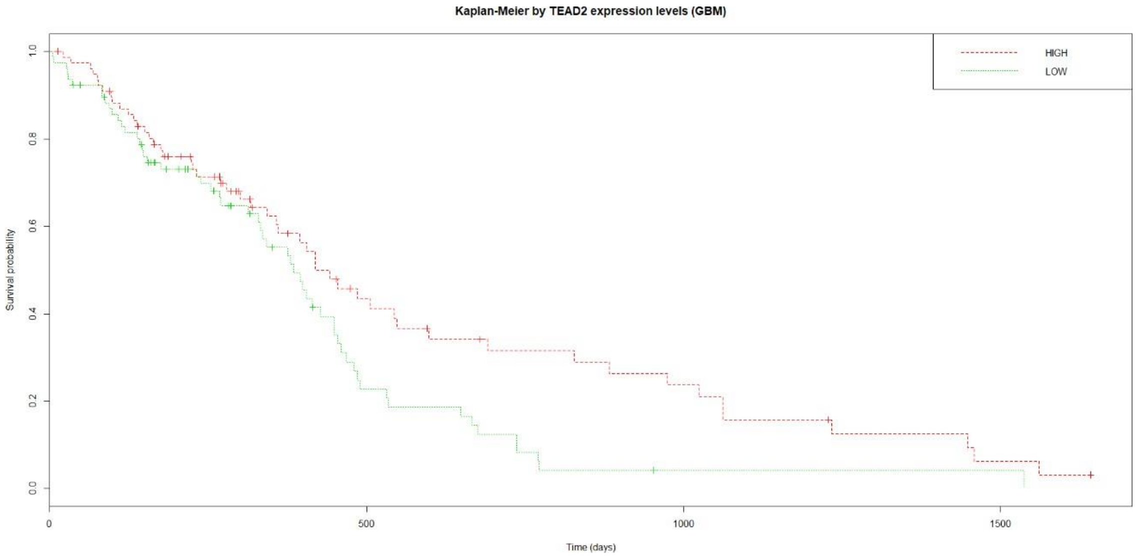

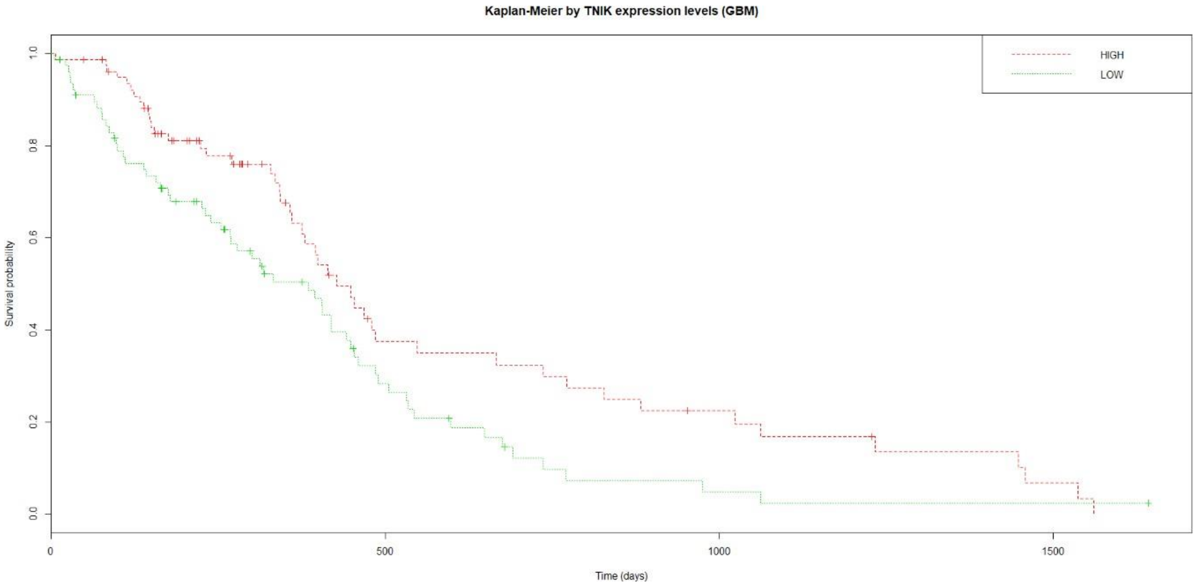

HNSC – Head and Neck Squamous Cell Carcinoma

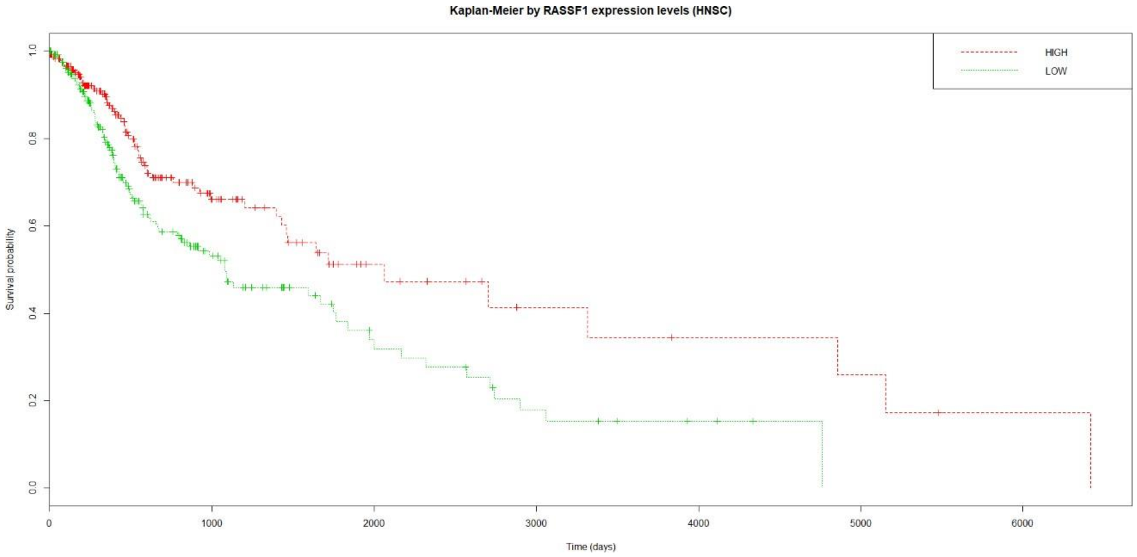

KIRC – Kidney Renal Clear Cell Carcinoma

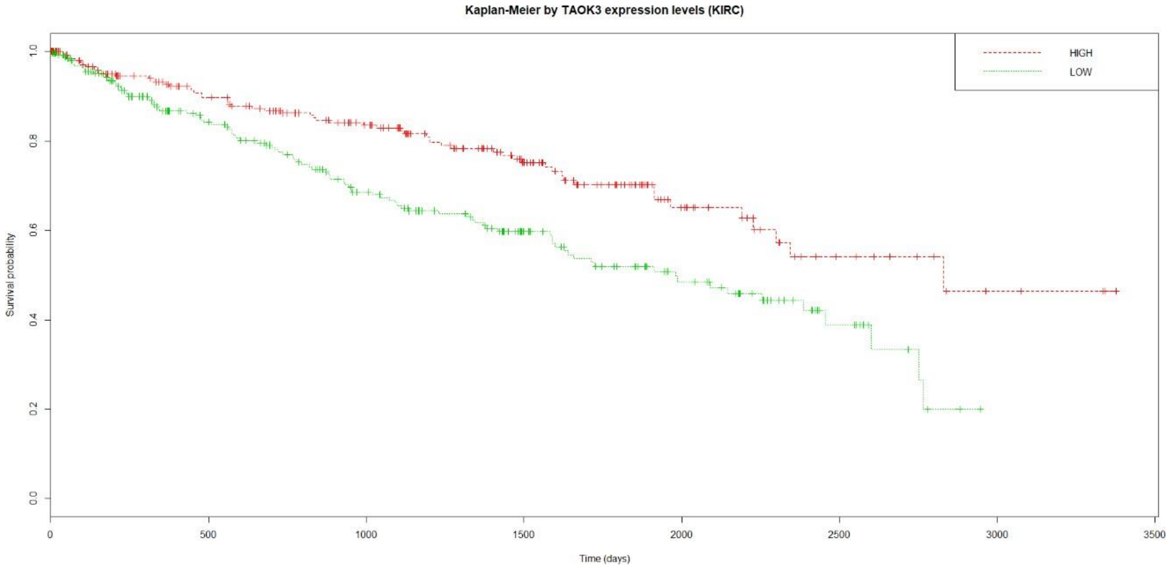

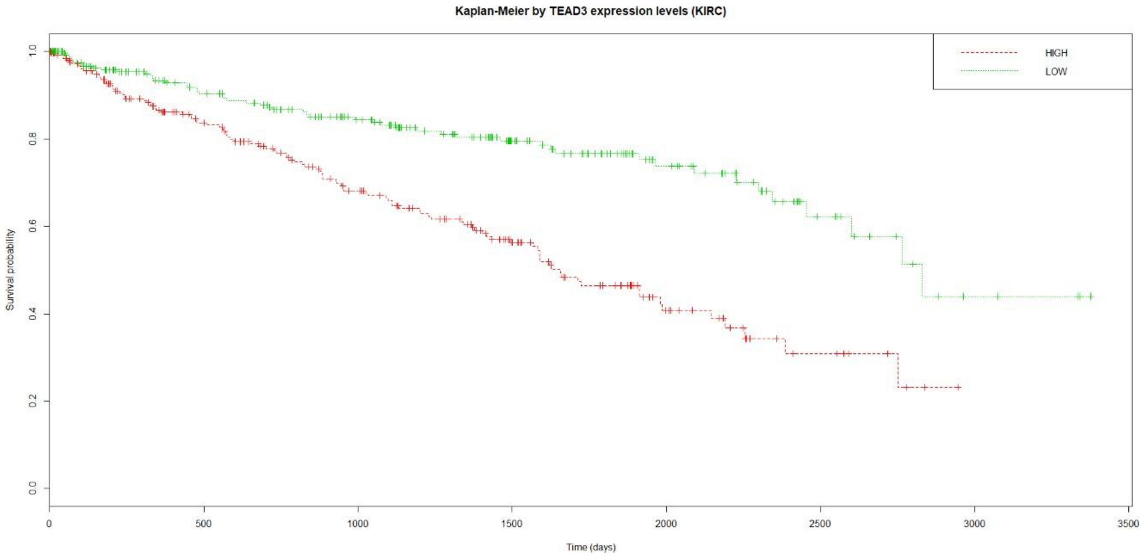

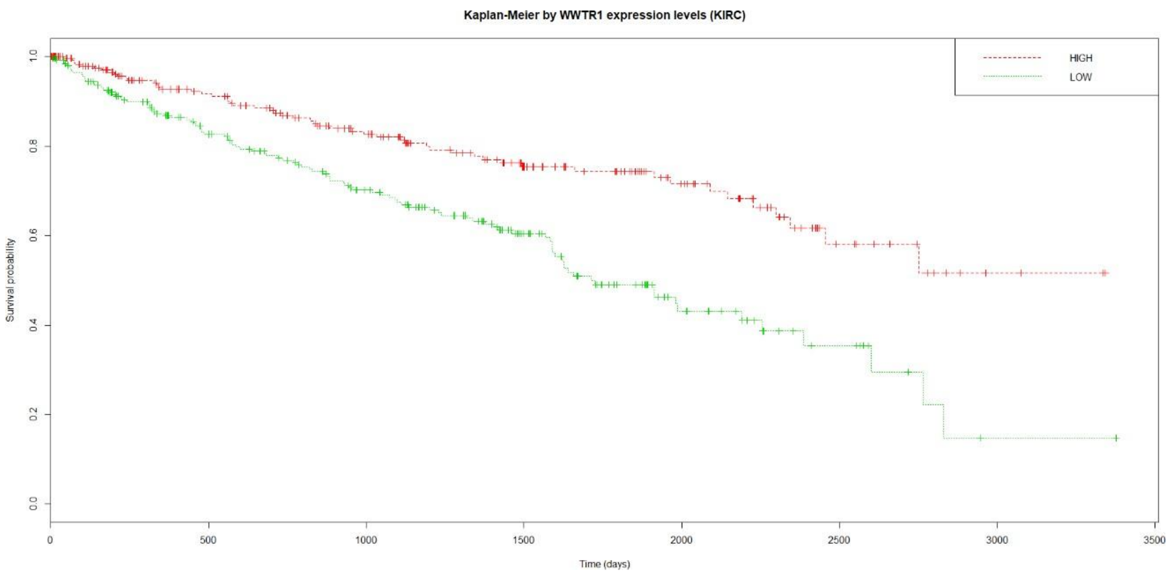
Kaplan-Meier by WWTR1 expression levels (KIRC)
HIGH
LOW
Survival probability
Time (days)
0.0
0.2
0.4
0.6
0.8
1.0
0
500
1000
1500
2000
2500
3000
3500

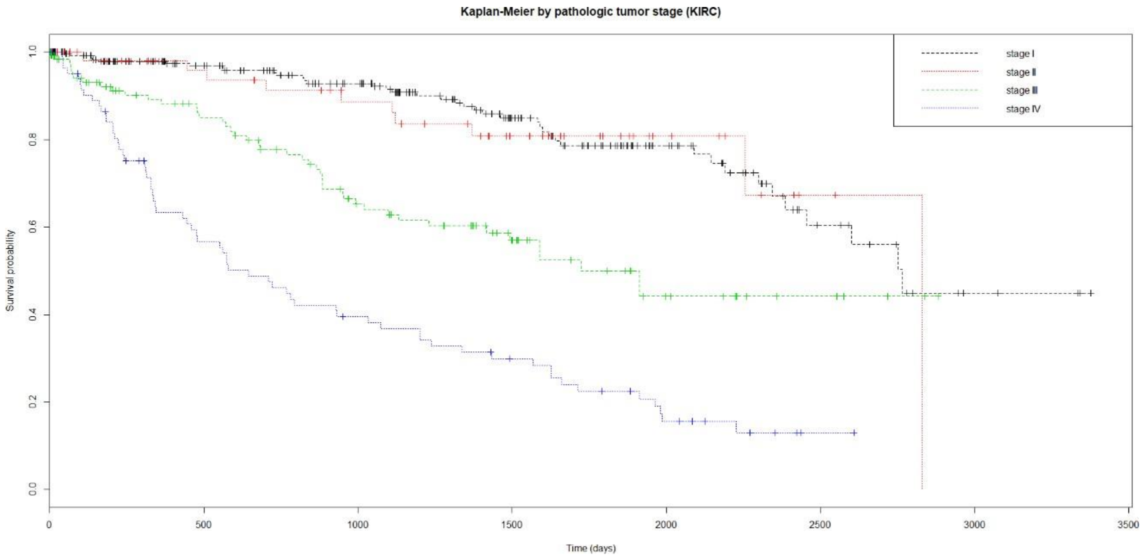
Kaplan-Meier by pathologic tumor stage (KIRC)
stage I
stage II
stage III
stage IV
Survival probability
Time (days)
0.0
0.2
0.4
0.6
0.8
1.0
0
500
1000
1500
2000
2500
3000
3500

LIHC – Liver Hepatocellular Carcinoma

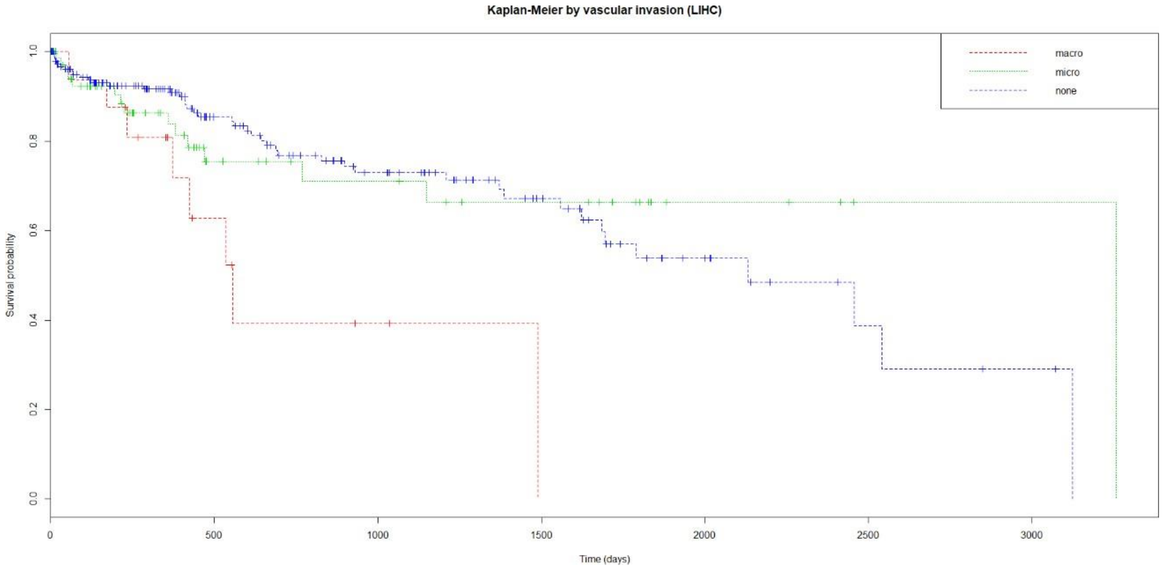

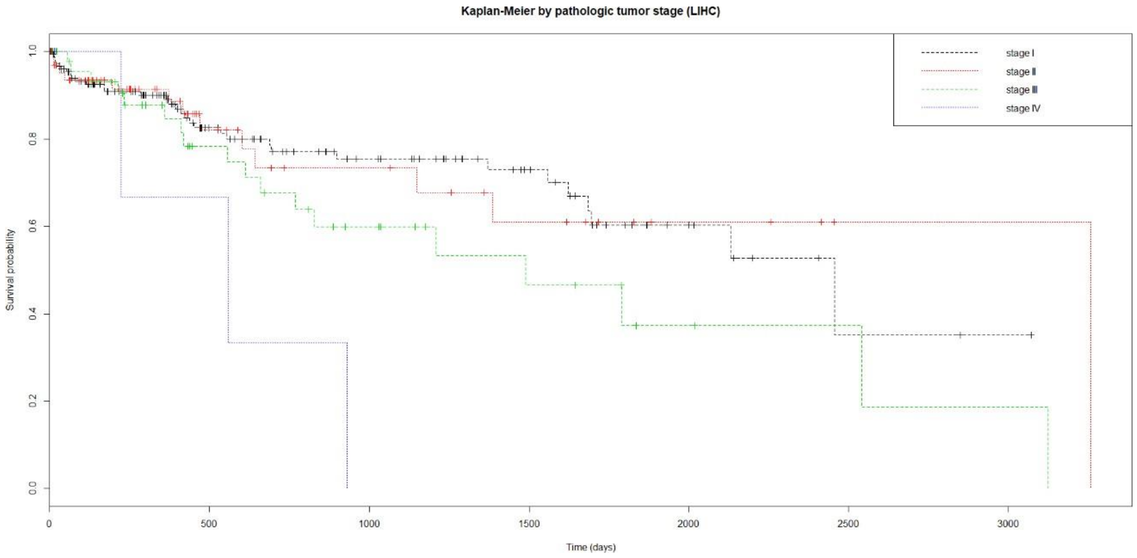

LUAD – Lung Adenocarcinoma

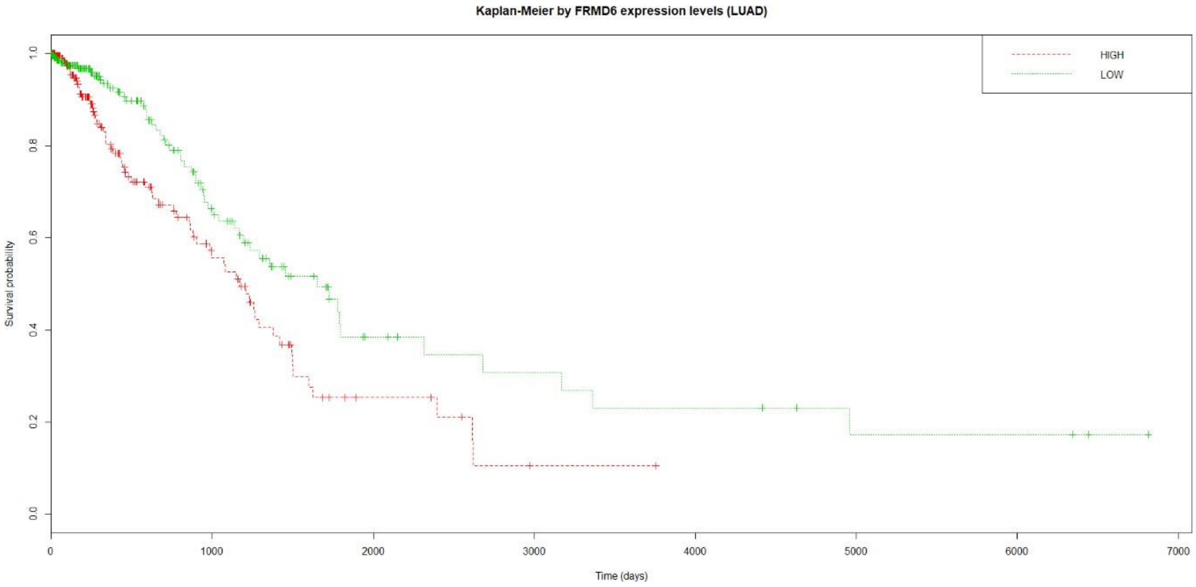

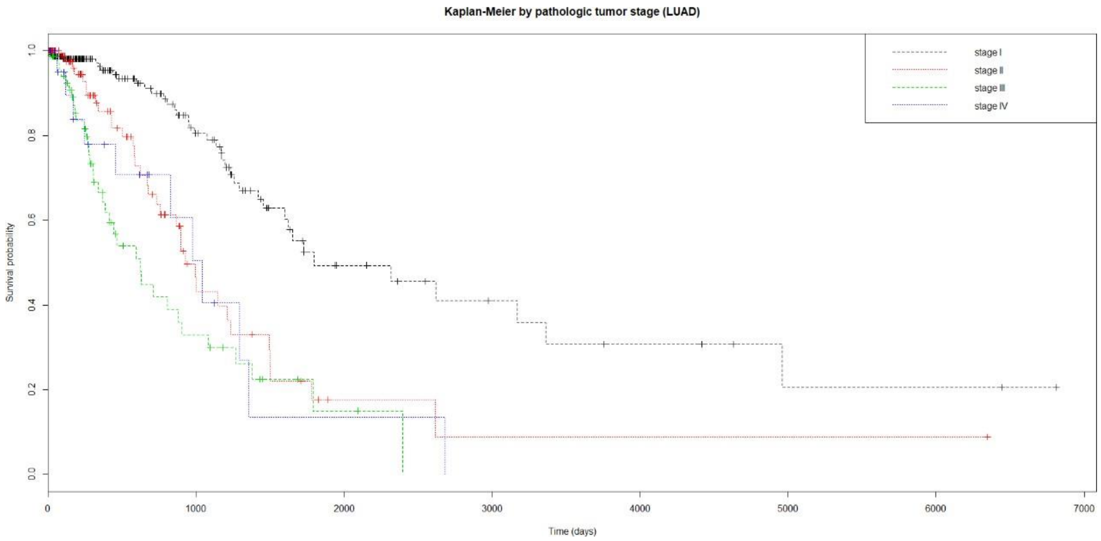

LUSC – Lung Squamous Cell Carcinoma

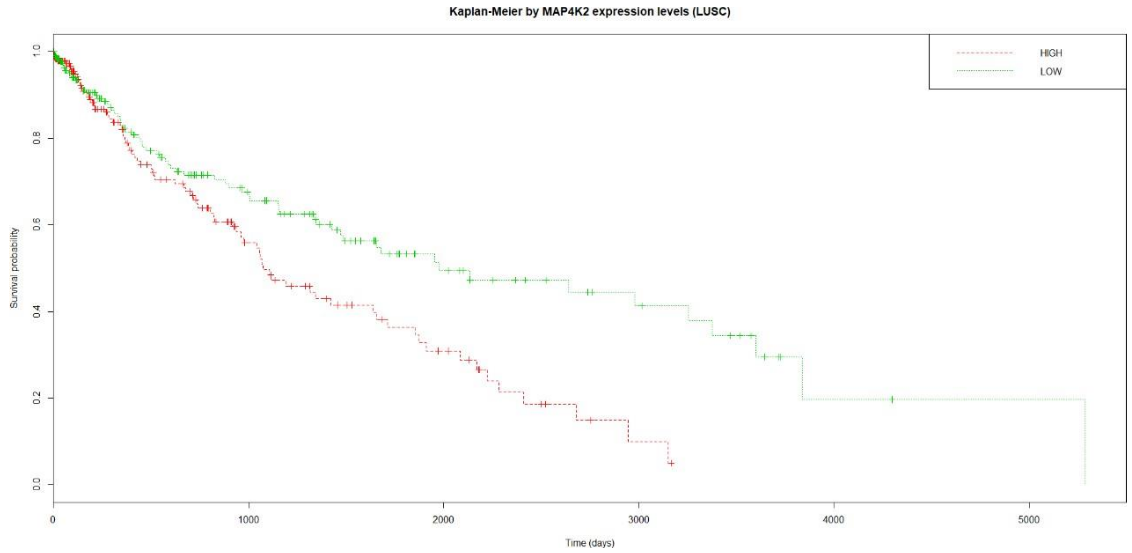

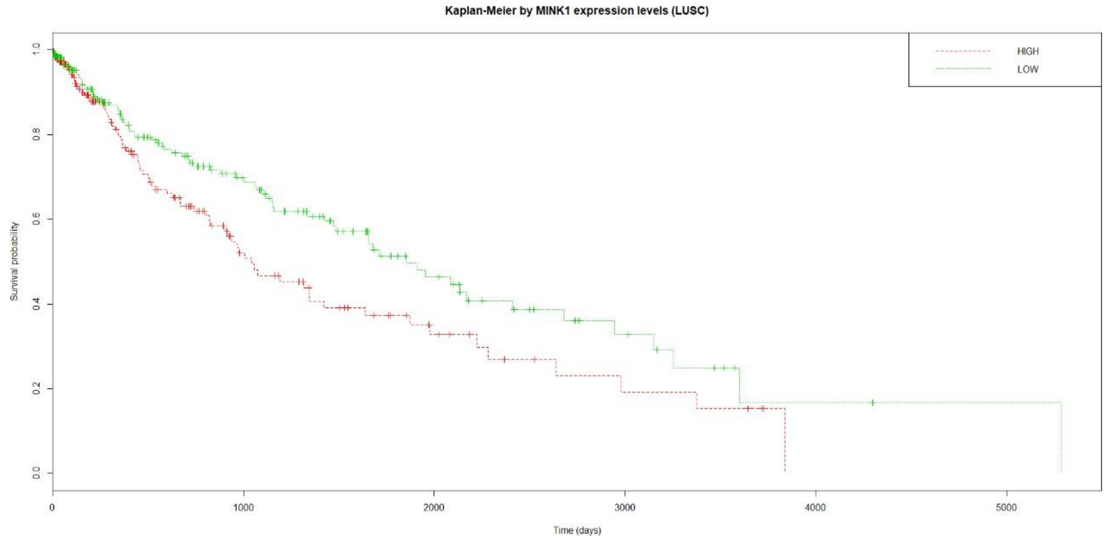

MESO – Mesothelioma

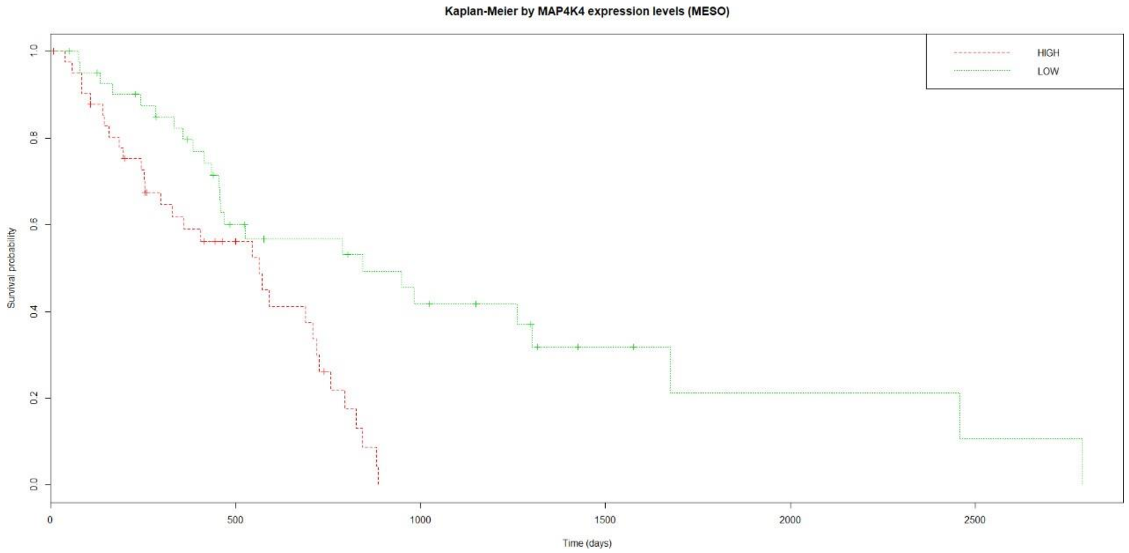

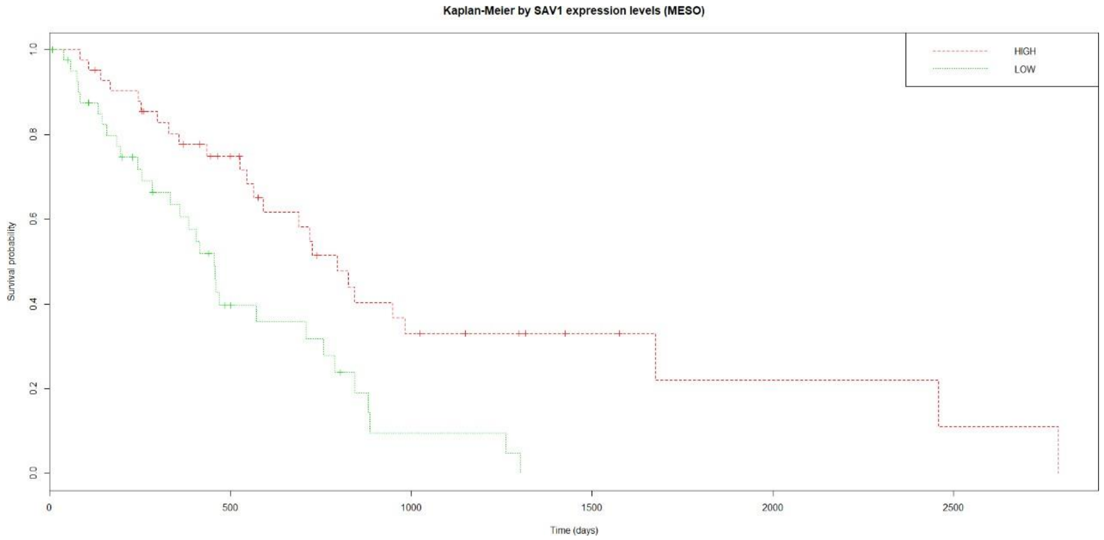

OV – Ovarian Serous Cystadenocarcinoma

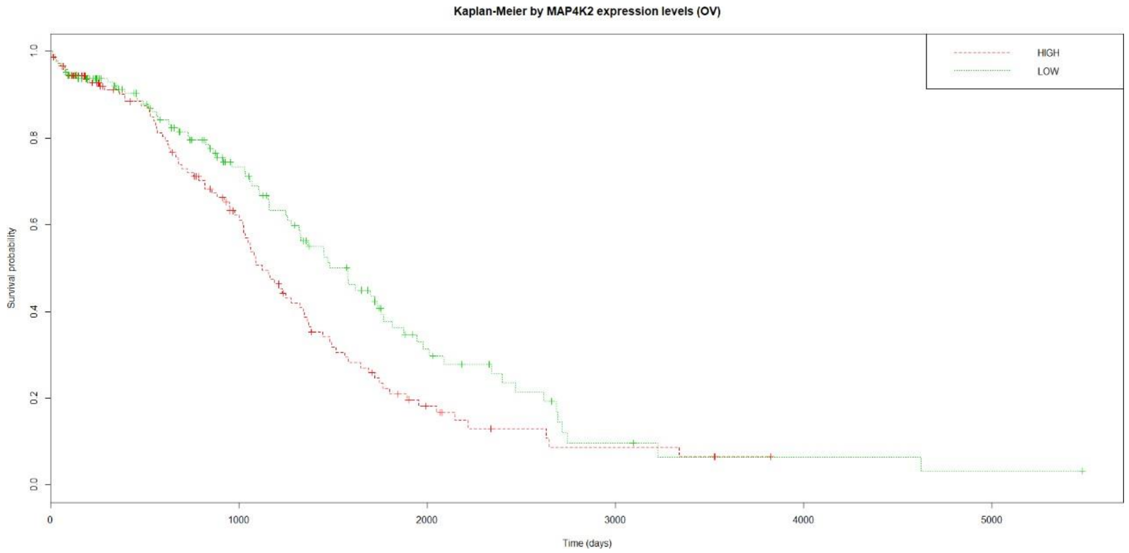

PAAD – Pancreatic Adenocarcinoma

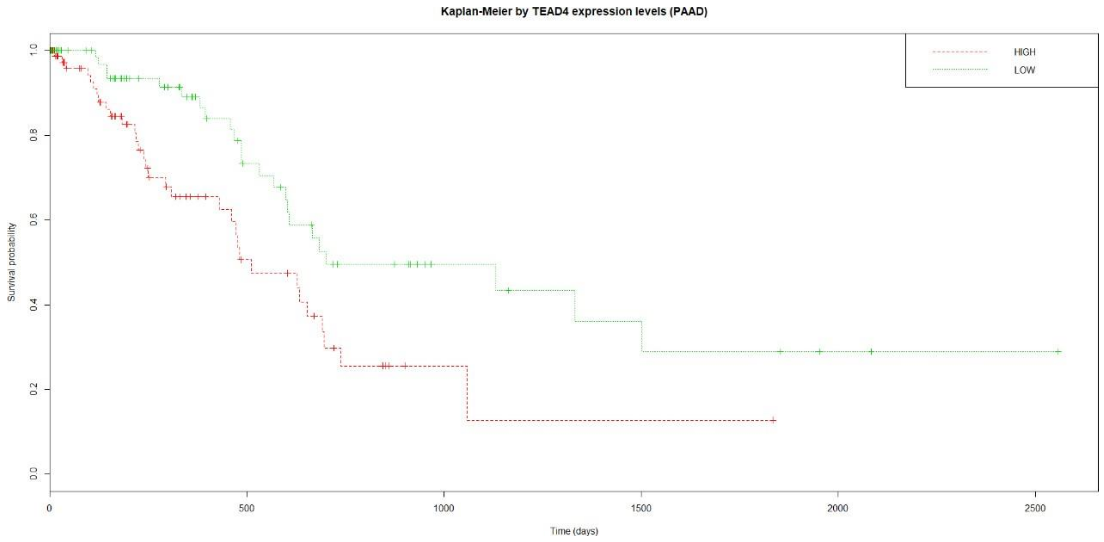

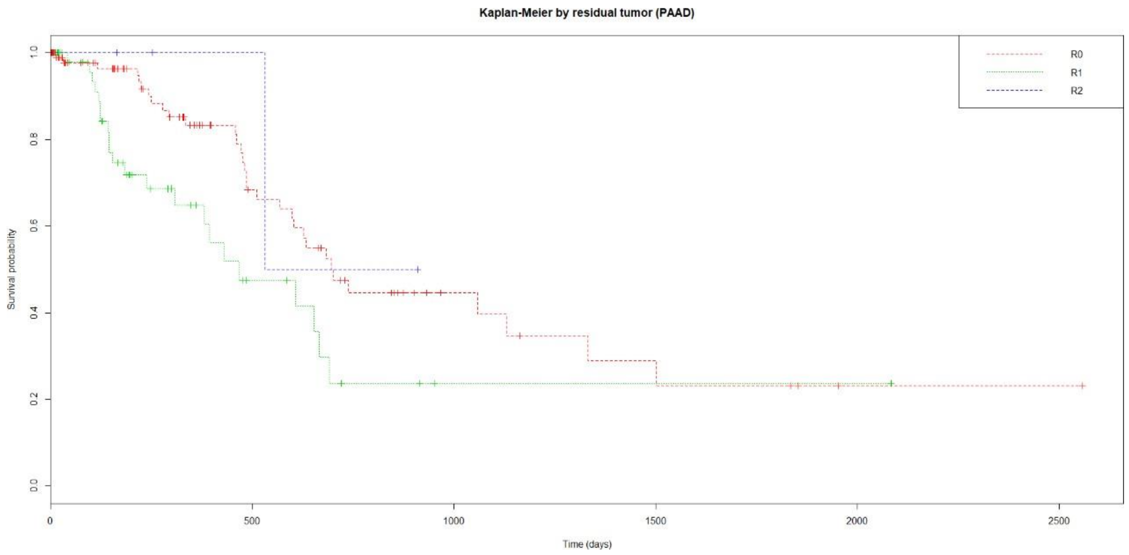

SKCM – Skin Cutaneous Melanoma

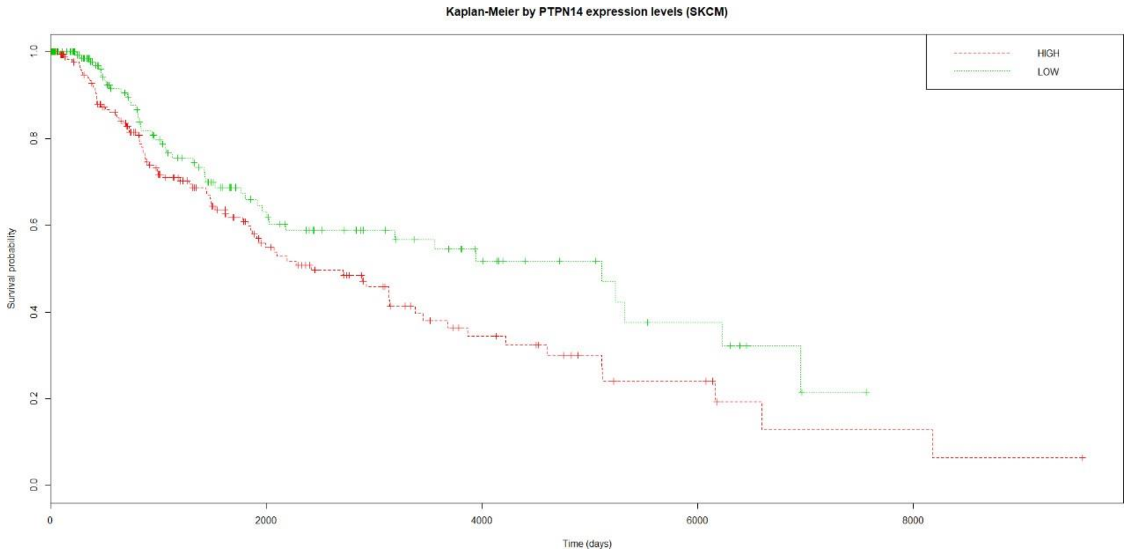

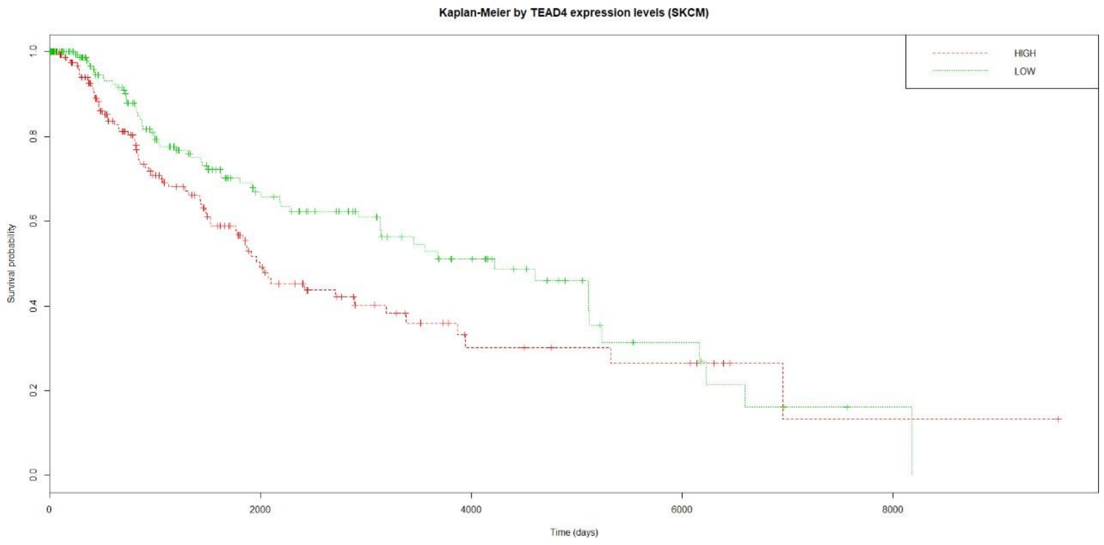

Supplementary figure S2. Correlation between independent predictors (mRNA levels of Hippo genes) and YAP1 or YAP1pS127 protein.

BLCA – Bladder Urothelial Carcinoma

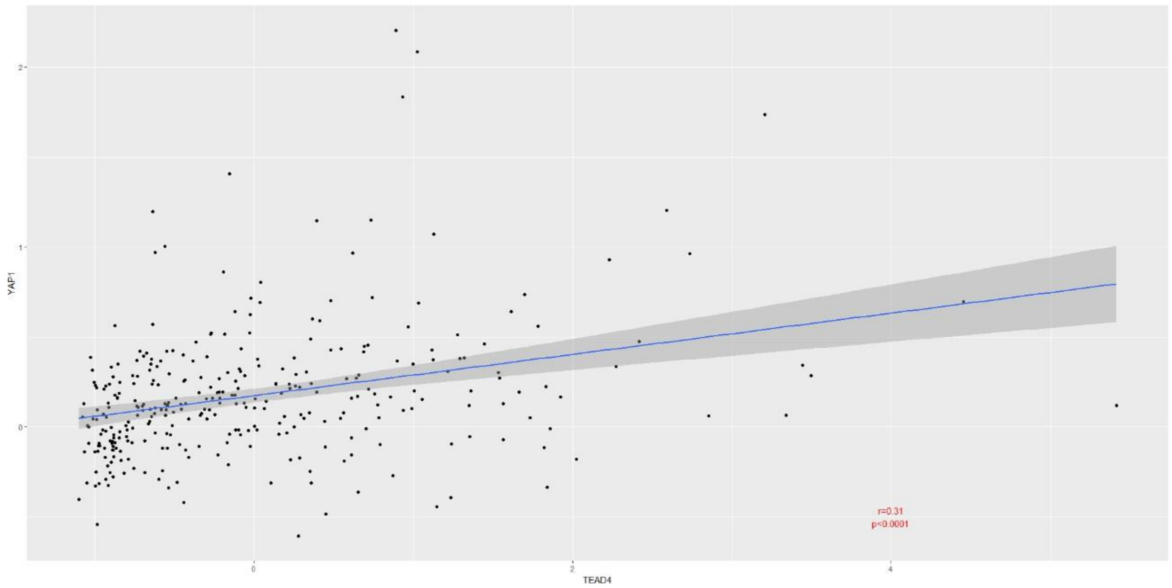

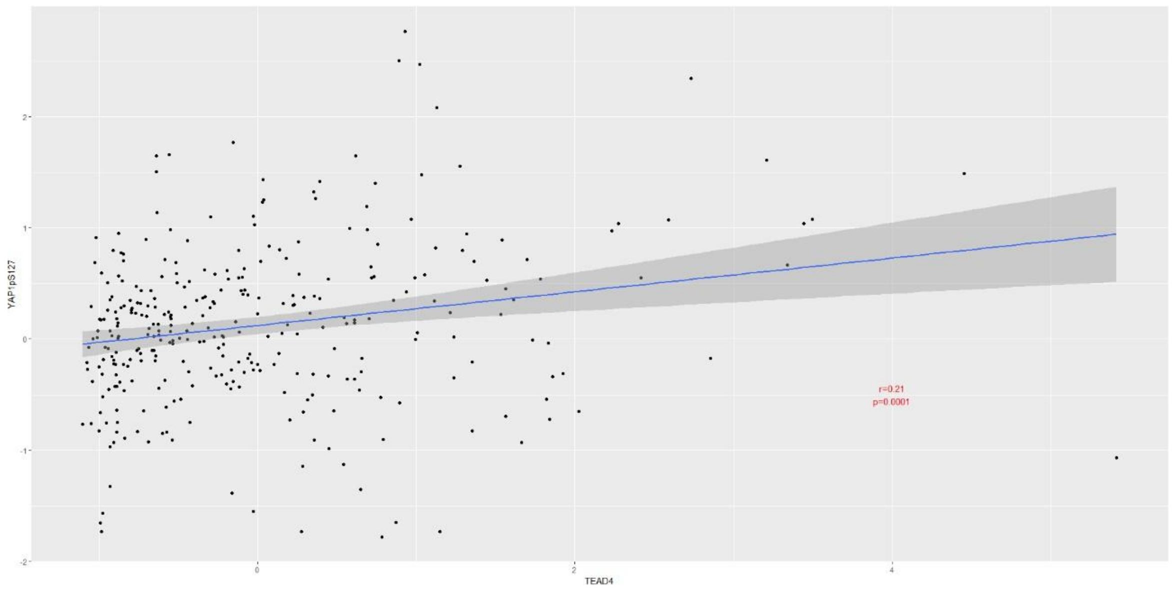

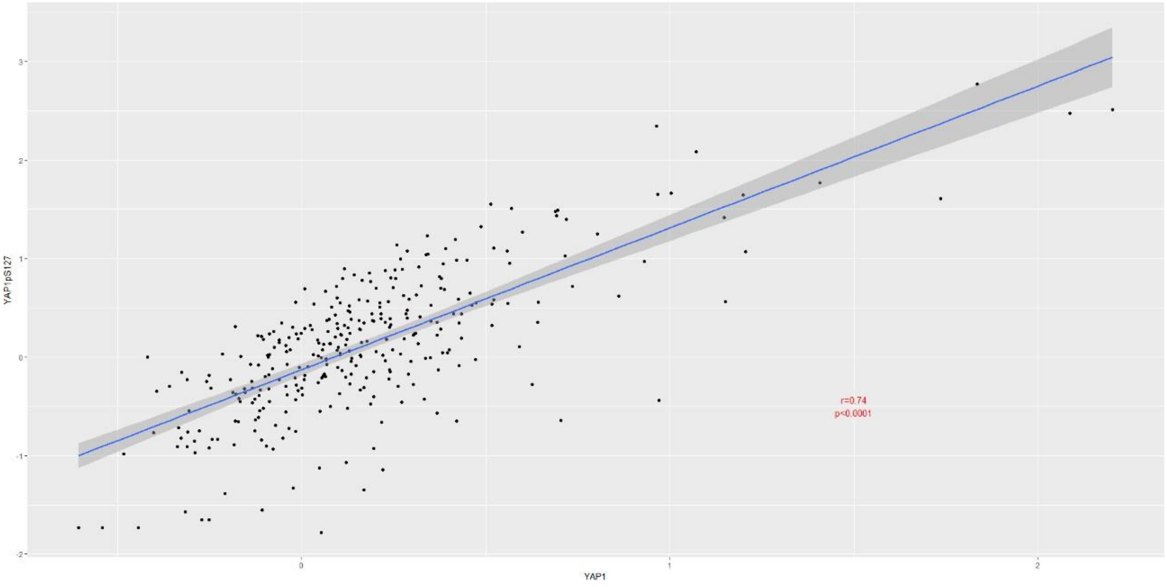

LGG – Brain Lower Grade Glioma

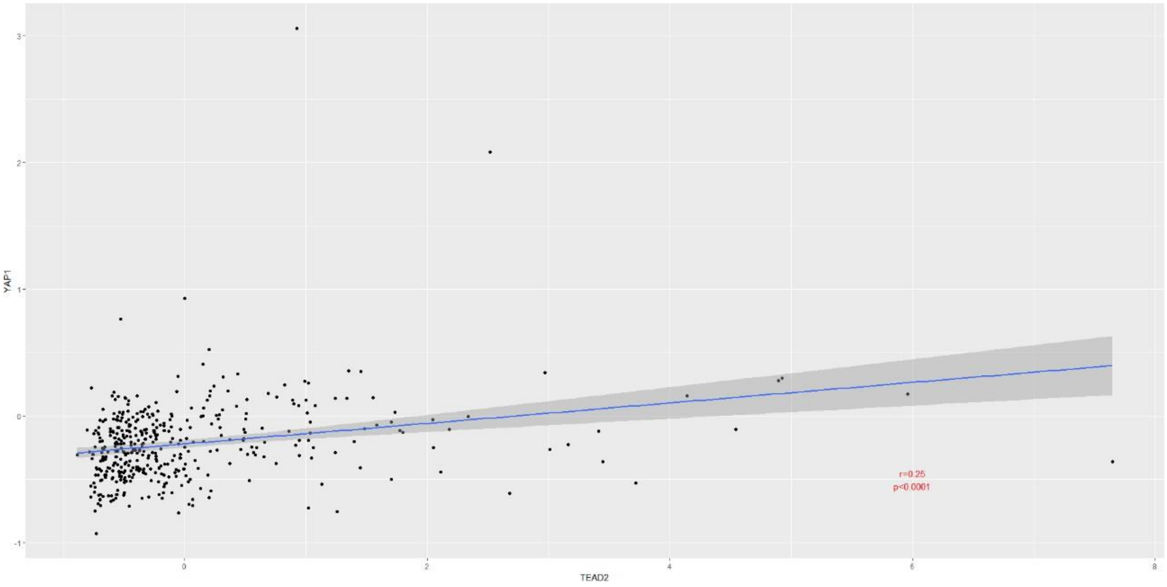

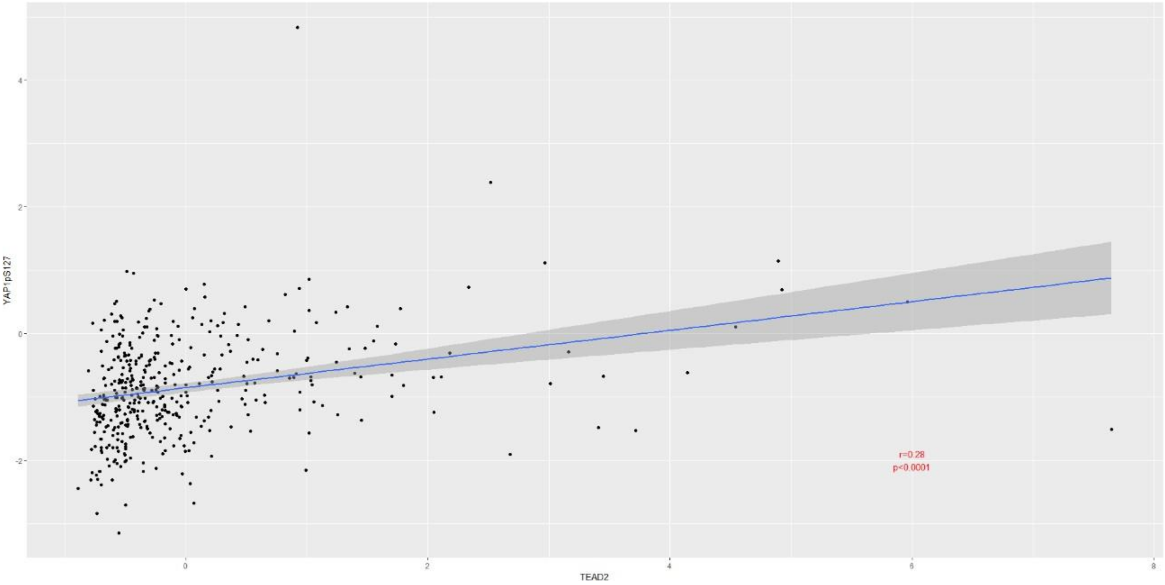
4
2
0
-2
YAP1pS127
0
2
4
6
8
TEAD2
r=0.28
p<0.0001

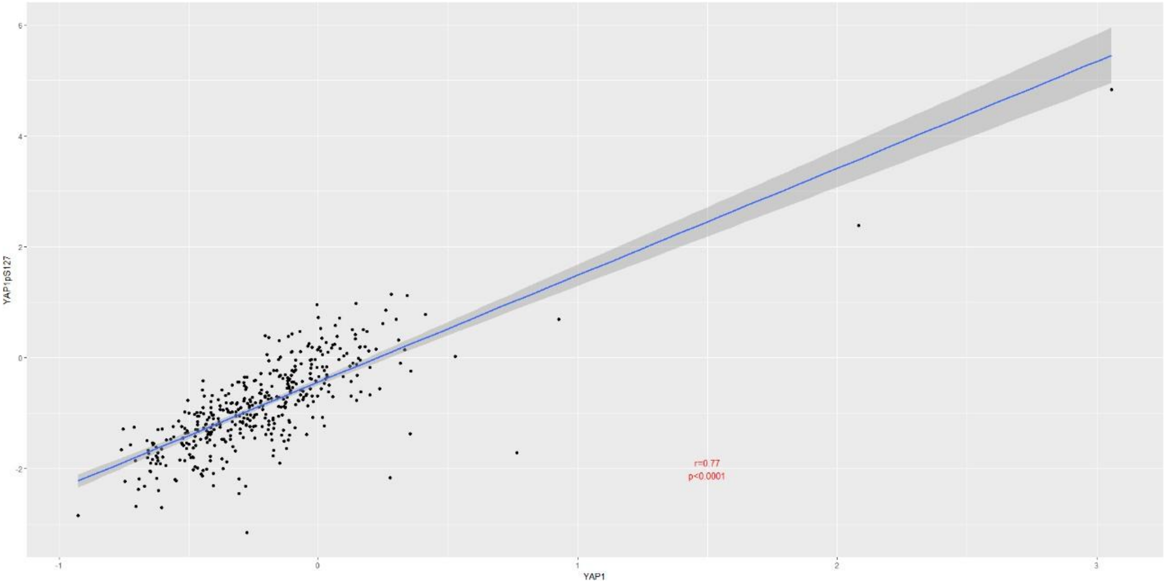
6
4
2
0
-2
YAP1pS127
-1
0
1
2
3
YAP1
r=0.77
p<0.0001

CESC – Cervical Squamous Cell Carcinoma and Endocervical Adenocarcinoma

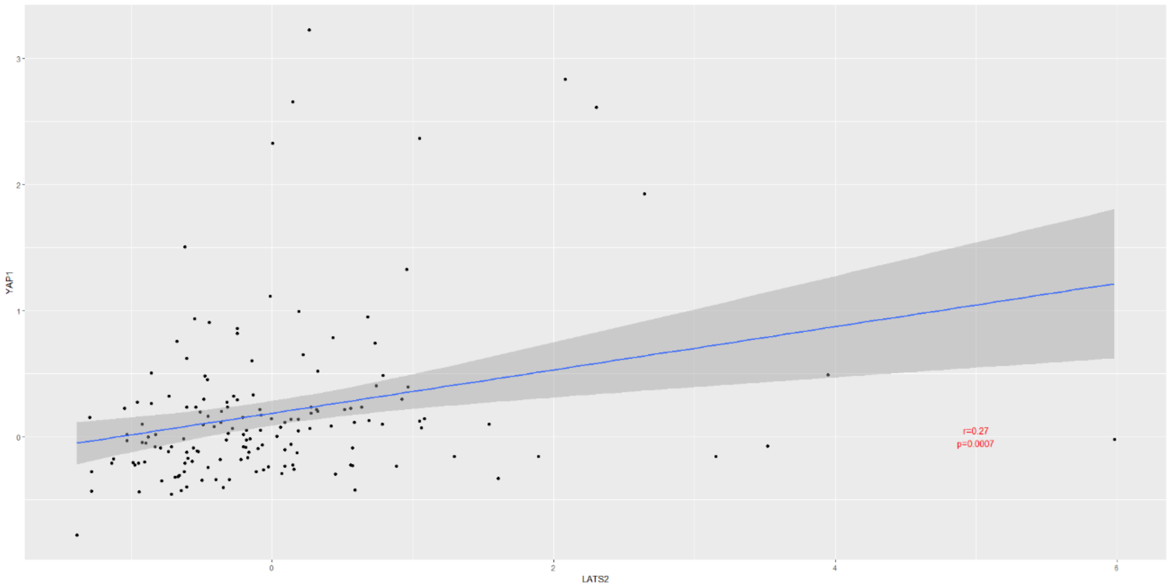

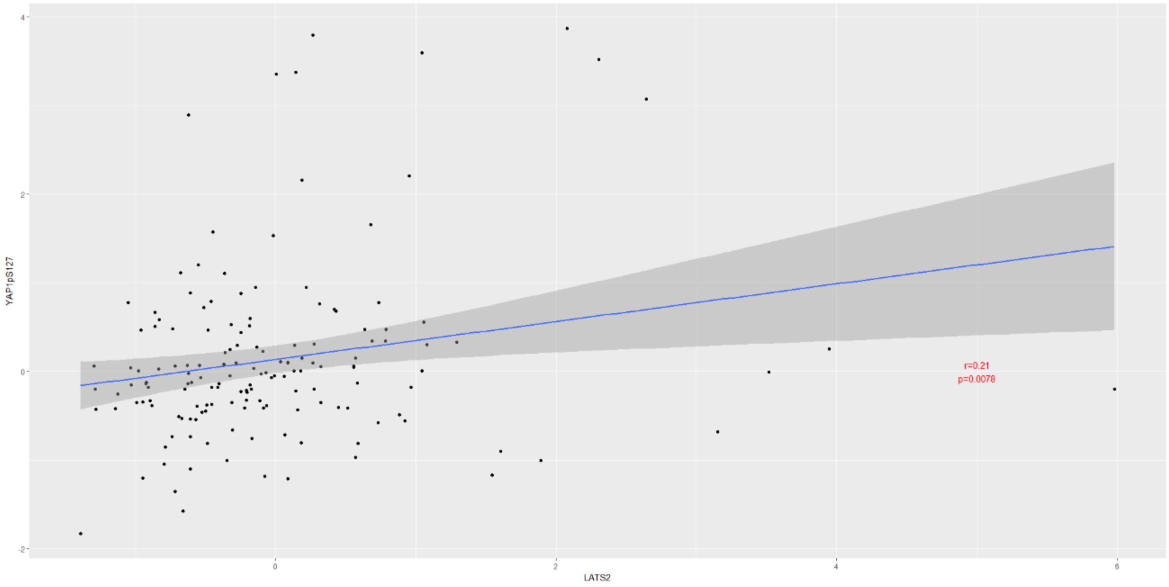

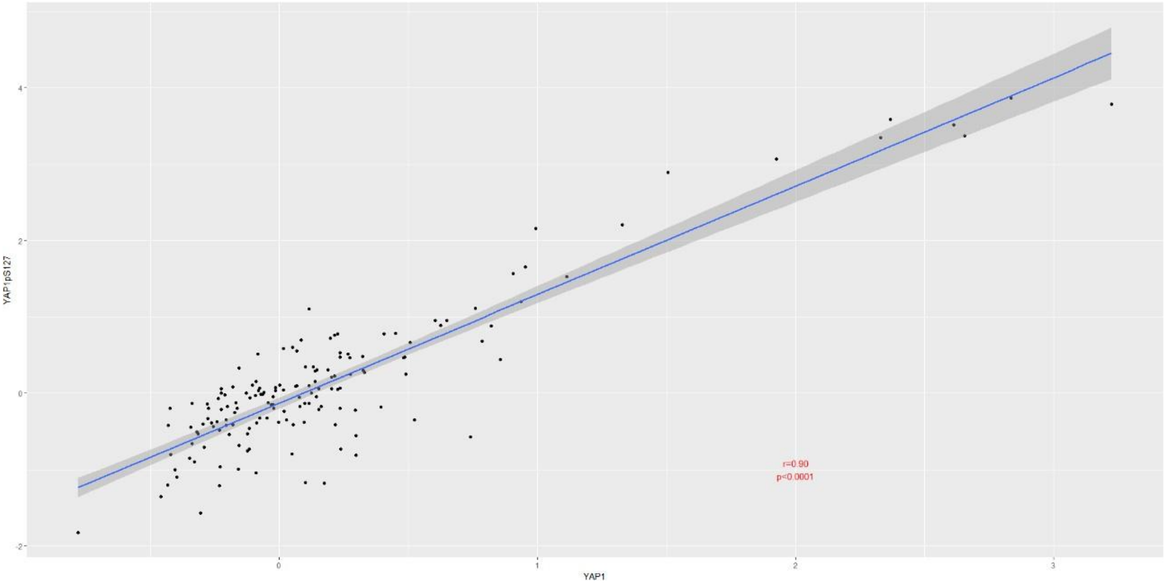

GBM – Glioblastoma

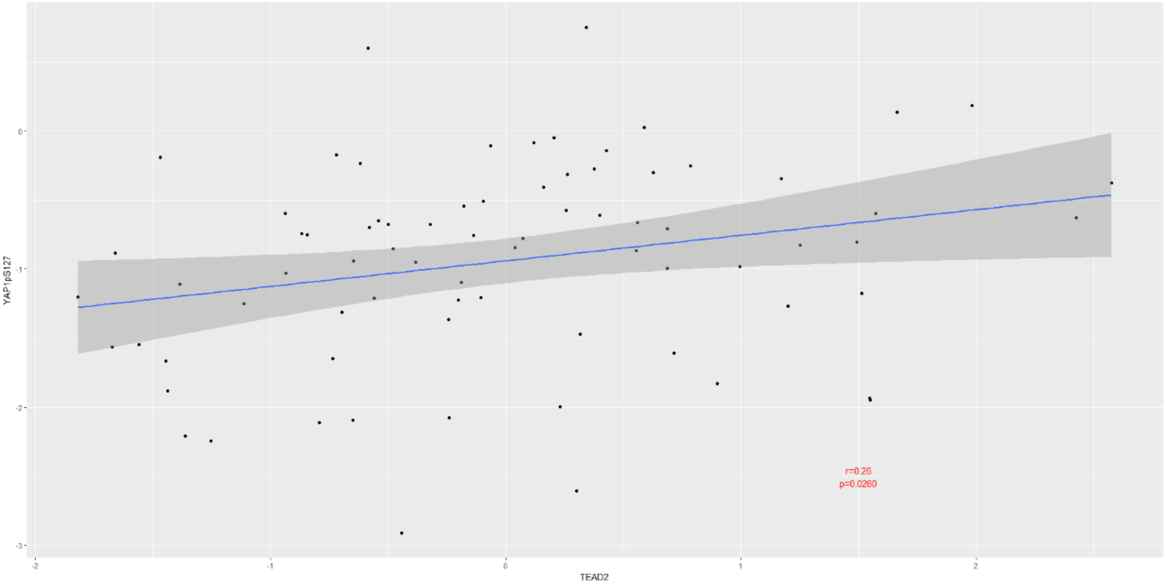

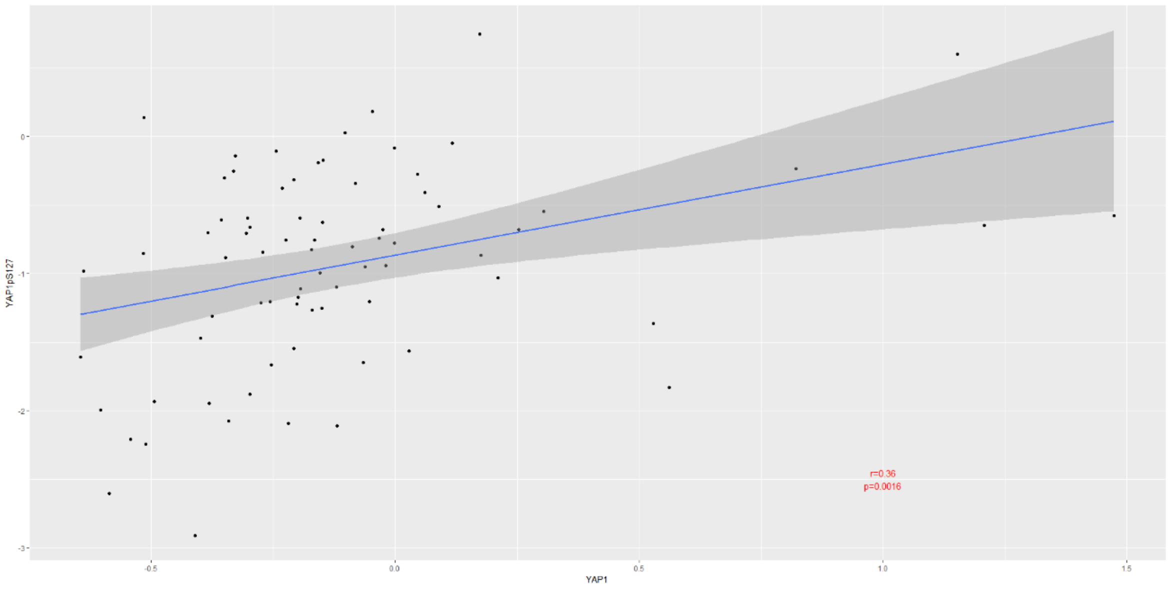

HNSC – Head and Neck Squamous Cell Carcinoma

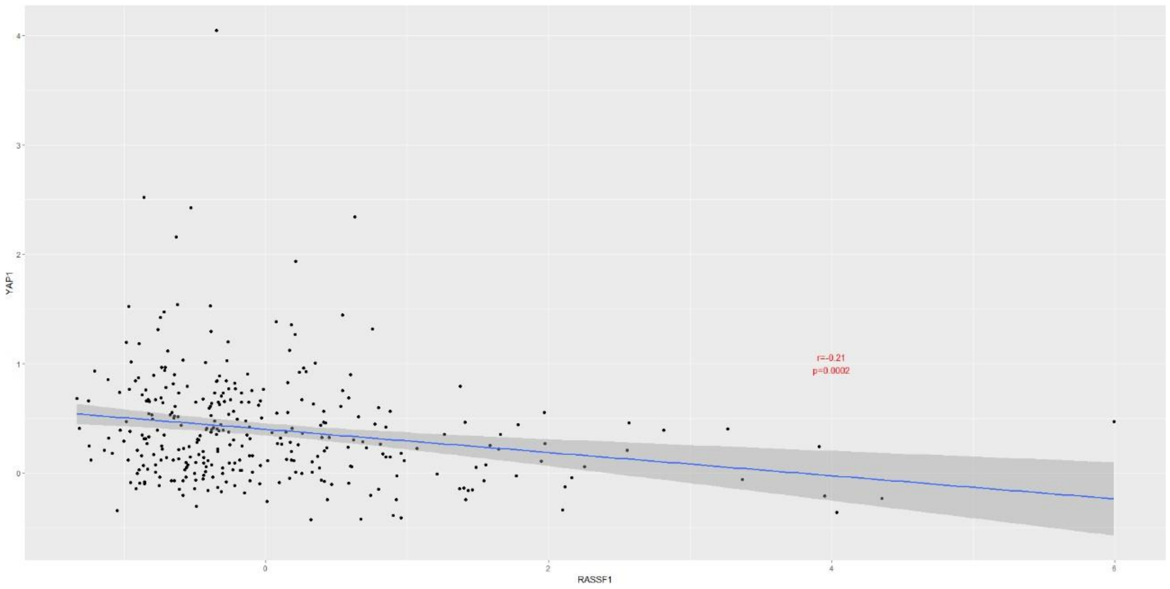

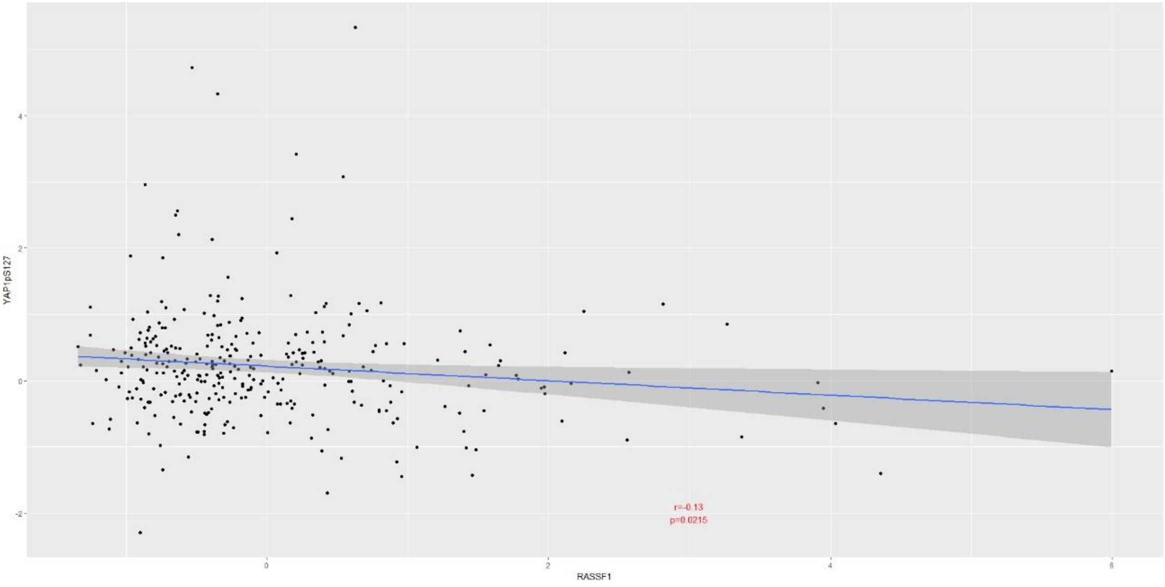
YAP1pS127
RASSF1
r=-0.13
p=0.0215

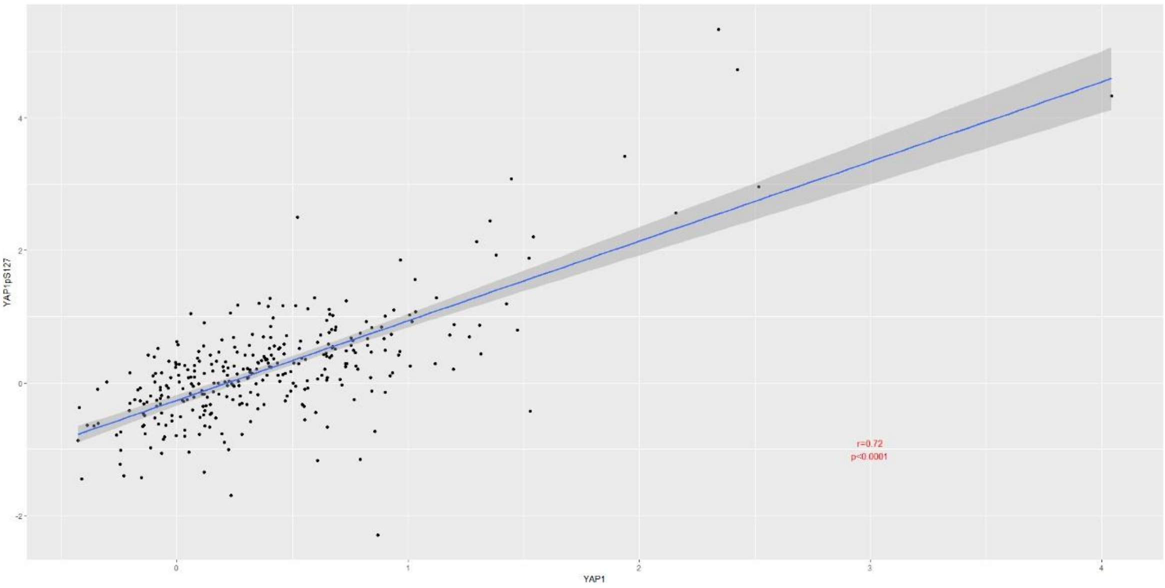
YAP1pS127
YAP1
r=0.72
p<0.0001

KIRC – Kidney Renal Clear Cell Carcinoma

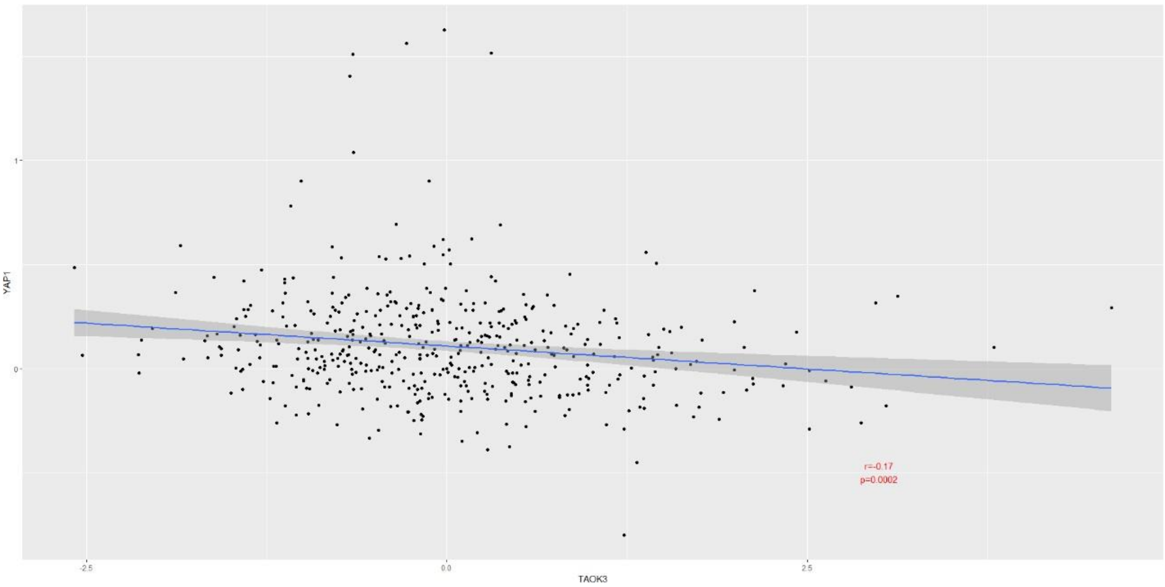

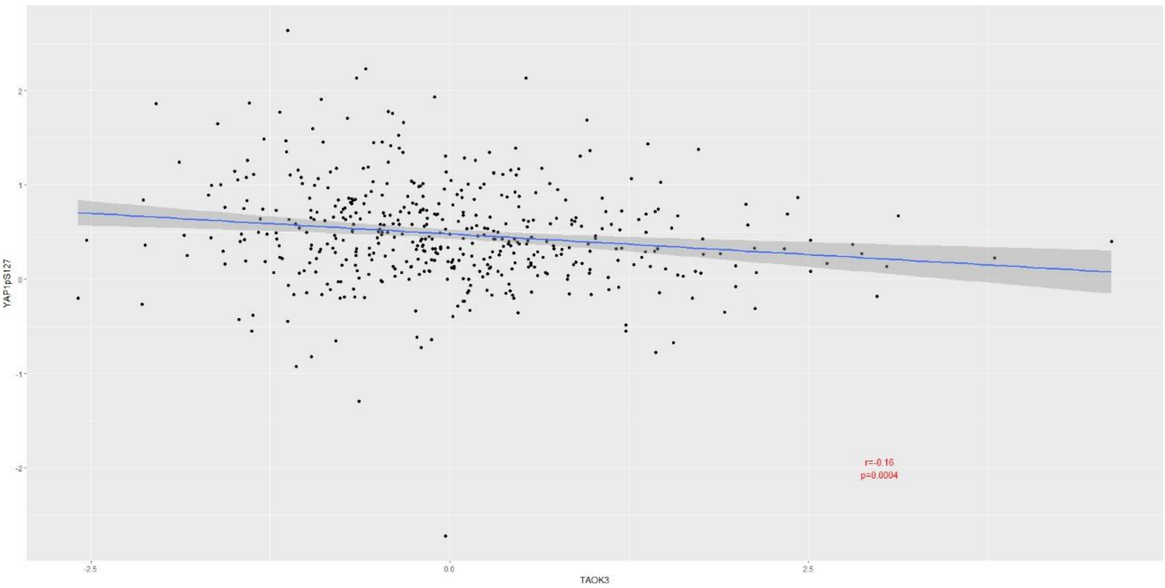

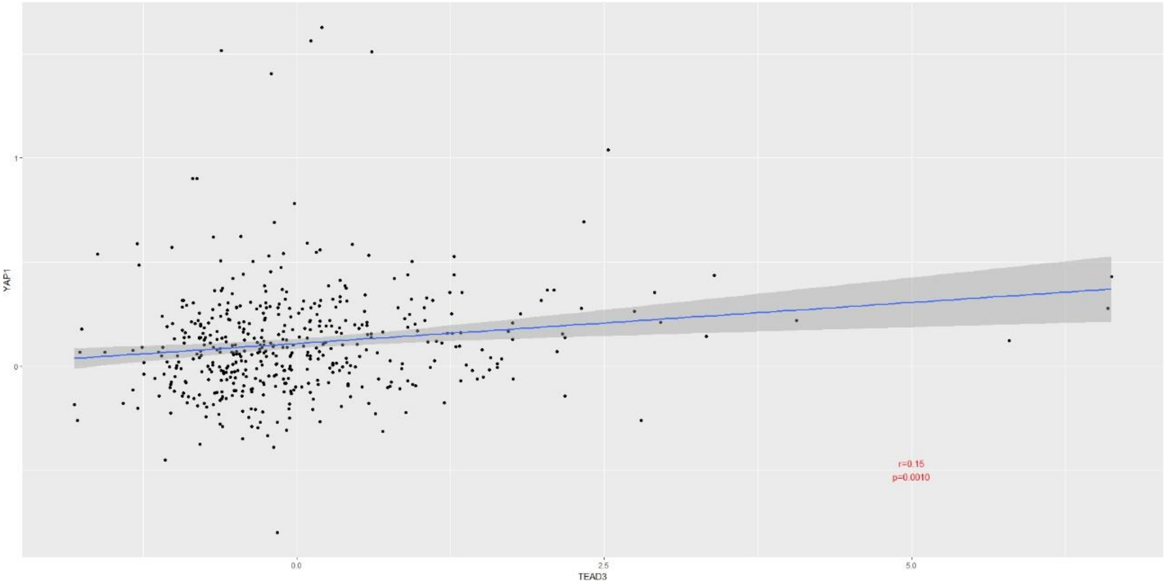
YAP1
TEAD3
1
0
0.0
2.5
5.0
r=0.15
p=0.0010

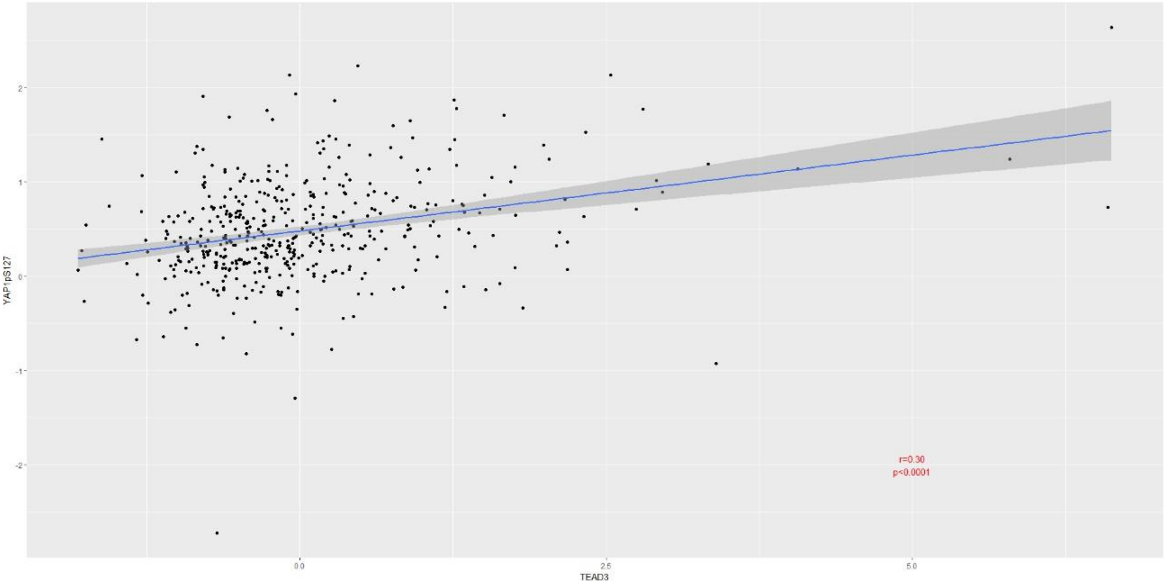
YAP1pS127
TEAD3
2
1
0
-1
-2
0.0
2.5
5.0
r=0.30
p<0.0001

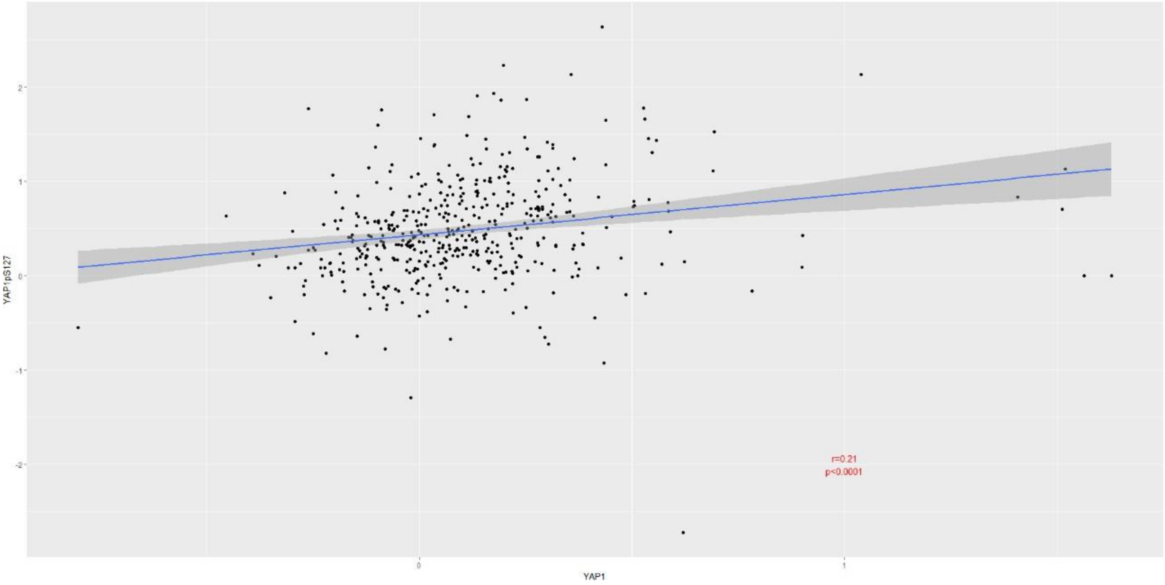
YAP1pS127
YAP1
2
1
0
-1
-2
0
1
r=0.21
p<0.0001

LUAD – Lung Adenocarcinoma

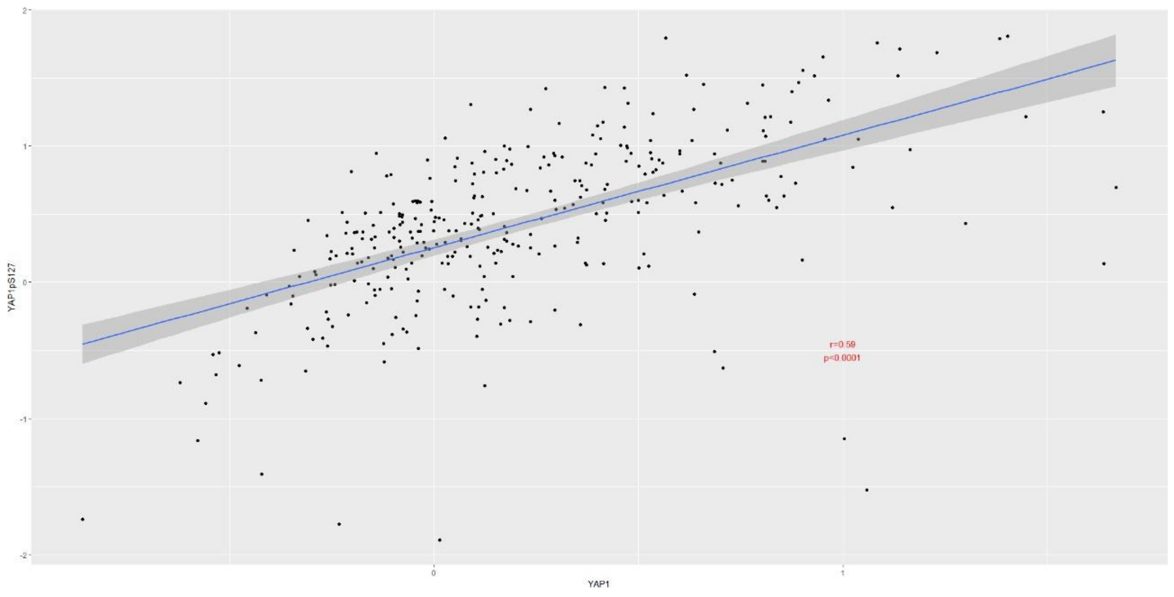

LUSC – Lung Squamous Cell Carcinoma

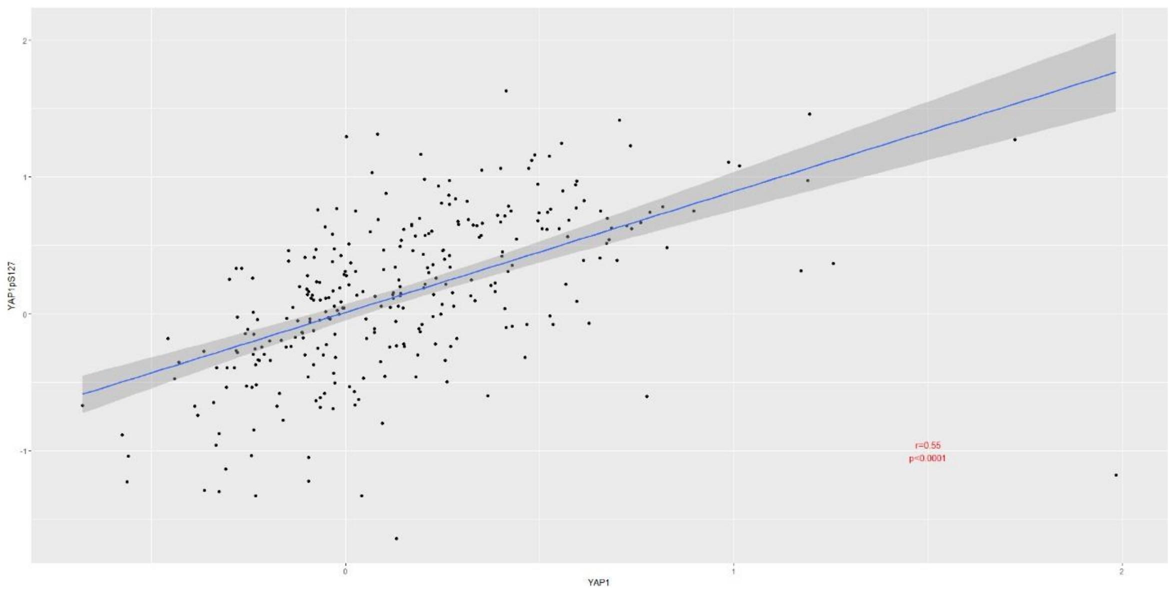

MESO – Mesothelioma

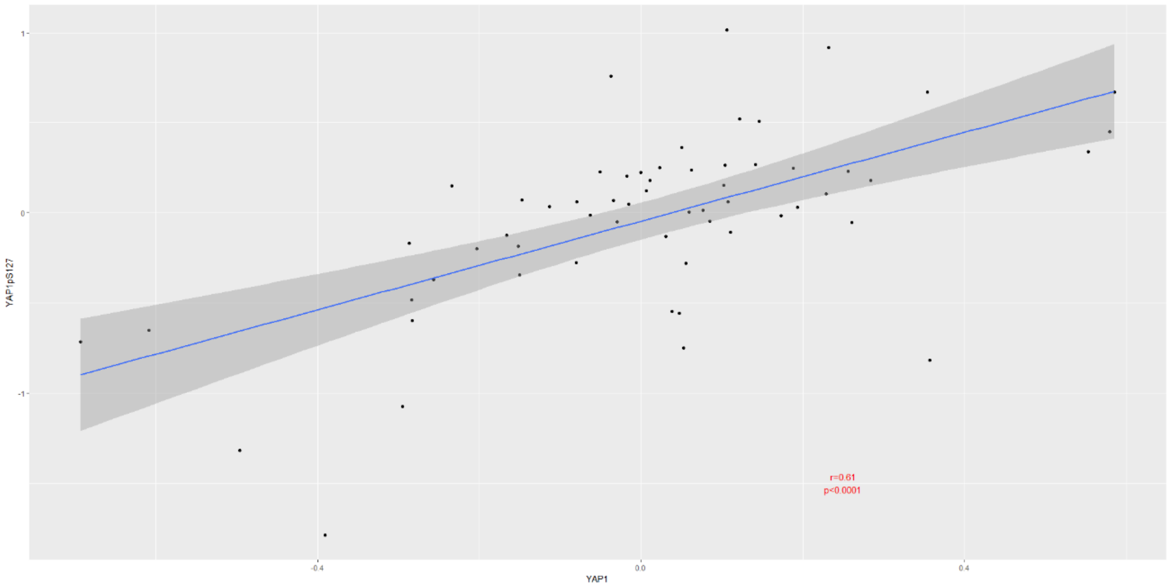

OV – Ovarian Serous Cystadenocarcinoma

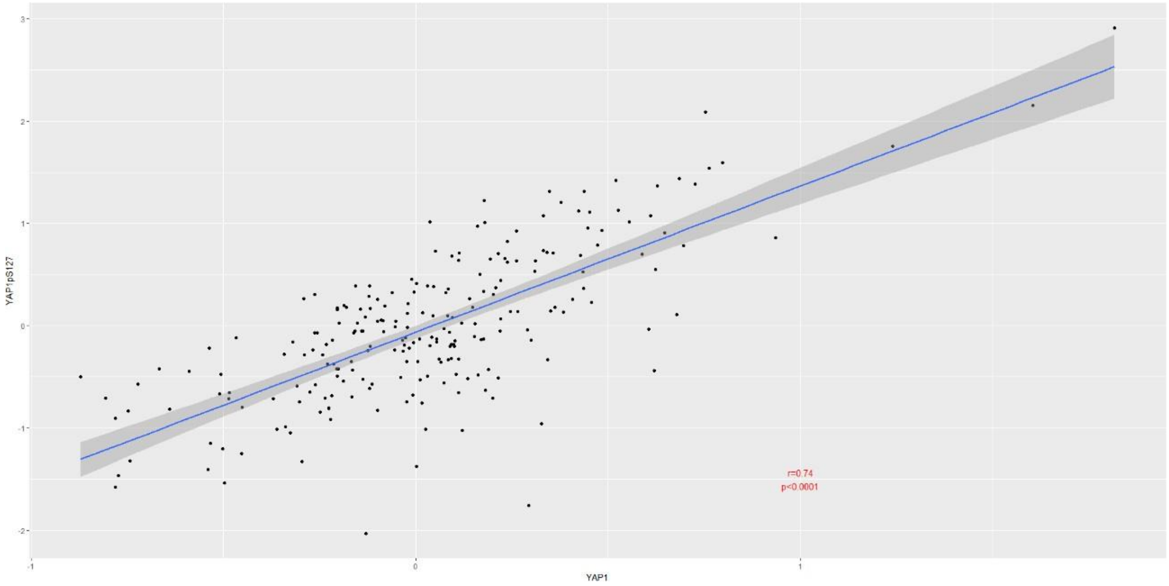

PAAD – Pancreatic Adenocarcinoma

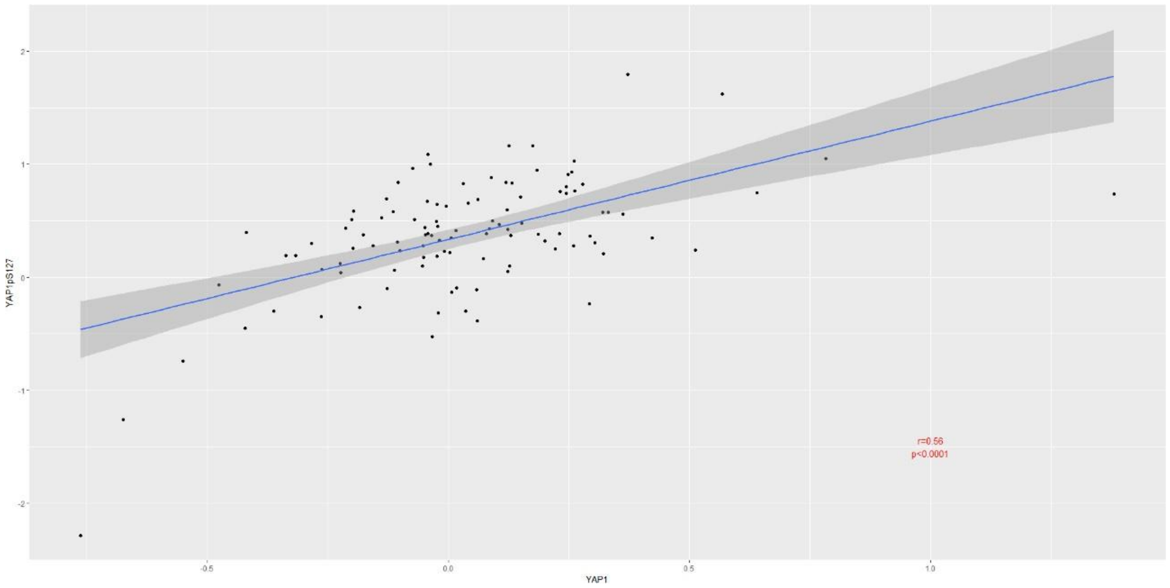

SKCM – Skin Cutaneous Melanoma

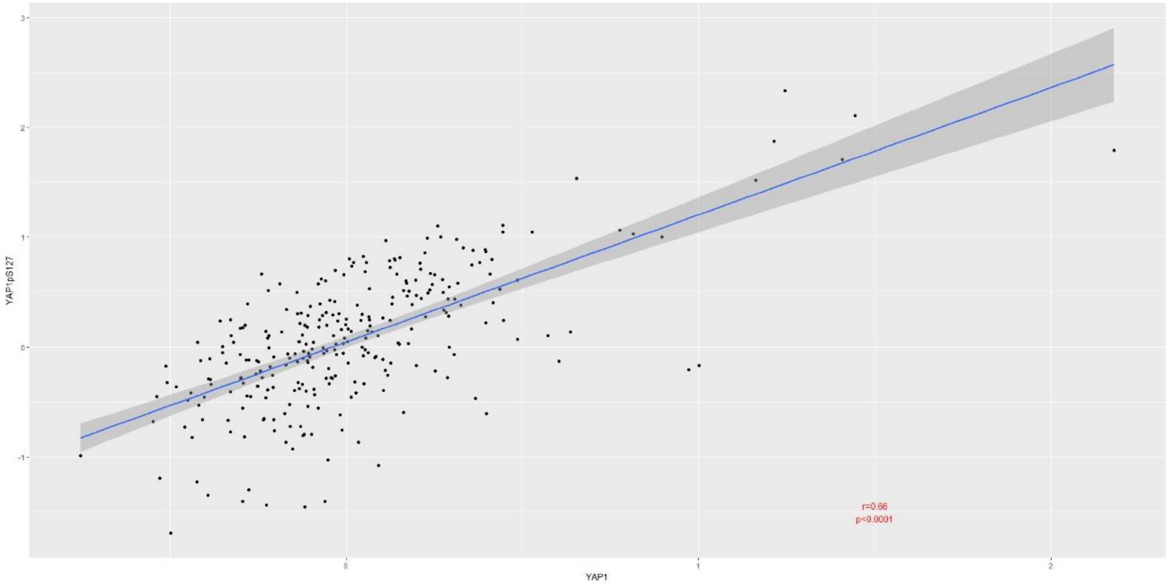

Supplement: Supplementary file 1 — Supplementary Information [file 41598_2018_28928_MOESM1_ESM.pdf]
